# Supplementary material for: Pytheas: a software package for the automated analysis of RNA sequences and modifications via tandem mass spectrometry
Source: Nat Commun. 2022 May 3;13:2424. doi: 10.1038/s41467-022-30057-5 (PMC9065004; doi:10.1038/s41467-022-30057-5)
Supplement: Supplementary file 1 — Supplementary Information [file 41467_2022_30057_MOESM1_ESM.pdf]

SUPPLEMENTARY INFORMATION

Pytheas: a software package for the automated analysis of RNA sequences and modifications via tandem mass spectrometry

Luigi D'Ascenzo[1,2,#,*], Anna M. Popova[1,#,*], Scott Abernathy[3], Kai Sheng[1], Patrick A. Limbach[3] and James R. Williamson[1,*]

[1] Department of Integrative Structural and Computational Biology, The Scripps Research Institute, La Jolla, CA, USA

[2] Current address: Department of Structural Biology, Genentech Inc., South San Francisco, CA, USA

[3] Rieveschl Laboratories for Mass Spectrometry, Department of Chemistry, University of Cincinnati, PO Box 210172, Cincinnati, OH, USA

[#] Authors contributed equally to this work: Luigi D'Ascenzo, Anna M. Popova

* Corresponding authors.

Corresponding authors contact information:

Luigi D'Ascenzo: dascenzo.luigi@gene.com, Anna M. Popova: popova@scripps.edu, James R. Williamson: jrwill@scripps.edu

Supplementary Note 1

Additional details on Pytheas matching algorithm and the scoring function.

Pytheas S_p score is a numerical value computed for each oligonucleotide-spectrum match (OSM) and represents the confidence in the sequence assignment based on the quality of the fit between theoretical and experimental MS/MS spectra. The output of the matching and scoring tool in Pytheas contains lists of OSMs that are grouped by the precursor *m/z* and RT (retention time), sorted in the descending S_p order, and assigned rank (**Supplementary Fig. 12**). ΔS_p is an additional score that is calculated to measure the relative distance between the top and other competing OSMs. Two additional parameters in this work, ΔS_p2 and ΔS_pD, are used to determine how well the scoring function discriminates between rank 1 OSM and the competing target and the decoy OSMs with lower ranks.

The optimized Pytheas S_p score represented by **Equation 1** in **Methods** consists of three separate terms**.** $\frac{\sum I_{\text{match}}}{\sum I_{\text{all}}}$ term defines the fraction of an experimental spectrum that can be assigned using L theoretically predicted MS2 ions. $\frac{n}{L}$ term defines the fraction of the predicted fragment ions that were identified in the spectrum. *n* and *L* counting of the sequence-defining MS2 ions is executed in a charge-independent manner. For example, matched c4(-1) and c4(-2) are counted as a single instance of c4 fragment ion, but c4(-1) and c4(-2) peak intensities equally contribute to $\sum I_{\text{match}}$ and $\sum I_{all}$. This was set up to alleviate the uncertainty present in the process of MS2 ion charge prediction.

To calculate $\sum I_{\text{match}}$, only intensities of the matched sequence-defining ions are used. The search for sequence-defining ions is executed after precursor ion peak (M) and precursor ion losses (e.g., M-P, M-H_2O, M-B etc.) peaks are assigned and excluded from search via the associated mass exclusion windows. All peak intensities are then normalized to the most intense peak present in the spectrum after the exclusion, and by default a relative 5% intensity threshold applied. $\sum I_{\text{all}}$ is then calculated by including all peak intensities in the specified *m/z* range (e.g., 300-2000) that remain after precursor ion and precursor ion losses exclusion and after relative intensity cutoff. This was set to increase sensitivity of the Pytheas score to the spectral features that are more likely represented by the sequence-defining ions. Furthermore, normalization to $\sum I_{\text{match}}$ introduces penalties for unassigned peaks in the MS/MS spectrum, which can arise due to chromatographic co-elution of RNA oligonucleotides or otherwise caused by instrument noise. This improves specificity of S_p for the correct identification. Critically, Pytheas does not recognize isotopic envelopes of the fragment ions and only monoisotopic peaks (m) contribute to $\sum I_{\text{match}}$, while m and m+1, m+2 etc. isotopologue peaks contribute to $\sum I_{\text{all}}$. We plan to address this issue in future releases to avoid penalties introduced to the S_p score by the lack of isotopologue peak assignments.

While $\frac{\sum I_{\text{match}}}{\sum I_{\text{all}}}$ and $\frac{n}{L}$ terms measure the goodness of the fit between theoretically predicted and experimentally observed spectra, term $\sum B_s$ is used to reward consecutively matched MS2 ions found in each series s. Number of RNA fragmentation series is user-defined, and depends on the dissociation method (e.g. CID vs HCD), ion polarity (negative vs positive), 5' and 3' -chemistry of RNA, and possibly other factors (examples in **Supplementary Fig. 9-11** and **18**). $\sum B_s$ term effectively contributes to discriminate between anagrams (oligonucleotide sequences with same nucleoside composition and precursor ion mass, but different positional order of nucleosides).

The extended definition of B is shown below:

$$B_s = \begin{cases} 0, & i_{\text{max}} = 0 \\ \sum_{i}^{i_{\text{max}}} k_i * \beta(1 + \alpha * (i - 1)), & i_{\text{max}} \neq 0 \end{cases}$$

Here, i represents the connectivity index for consecutive matches, and k_i is a number of matches with connectivity index i found across the ion series s. β is a basal reward value for occurrence of a consecutive match, α is a reward coefficient used to increase the reward for the second, third etc. consecutive matches. As an example, for the set of matched ions in the w-series (w1, w2, w3, w4, w6) connectivity index is defined as follows: w1 and w6 (i = 0), w2(i = 1), w3(i = 2), w4(i = 3).

The standard values of α and β parameters were set to 0 and 0.075 respectively during the process of S_p training and validation. However, α and β can be easily changed by the user at the matching and scoring step, for instance to improve identification coverage for long oligonucleotides or oligos within specified size ranges. In his study, α = 2 and β = 0.025 have been used interchangeably with the default values for the analysis of several datasets presented (see **Pytheas Database Search** in **Methods**). In general, setting α > 0 increases S_p scores for sequences that are >10 nt in size, and improves sensitivity of their identifications. On the other hand, very large α values (or a combination of small α and large β) will quickly reduce S_p scores for short oligonucleotides (3-5 nt in size). We found empirically that the usage of α = 2 and β = 0.025 introduced minimal sequence length biases (S_p values spread over a narrow 0-2 range), and slightly improved $\Delta S_p D$ distribution compared to the default α = 0 and β = 0.075 parameter values. As for the global identification coverage, no significant differences (5-10 % more IDs) were observed by using either of these two sets of parameters.

Supplementary Note 2

From peptide SEQUEST score to oligonucleotide Pytheas score.

In contrast to limited cleavage of the peptide backbone, CID of RNA leads to more complex fragmentation pattern, with 9-11 ion series consistently observed in the spectrum. We found that including all the series in the original SEQUEST score improved identification. This was particularly important for matching short (3-5 nt) and long (> 10 nt) sequences. In the former case, due to high repeatability of nucleoside units in the sequence, competition between candidate matches is very tight, and large number of identified MS2 ions becomes crucial for correct identification. In the latter case, reduced ionization efficiencies and complex isotopic envelopes makes finding all predicted fragment ions difficult (**Supplementary Fig. 18**). Identification of long RNA sequences is thus enabled through larger amount of MS2 ions present, and through utilizing B_s reward for consecutive matches. The major goal of the optimization process was to alleviate the length-dependence of S_p (the "roof"-like behavior, with lower scores observed for short and long oligomers) presented by the original SEQUEST score, while retaining high specificity for correct identification.

Supplementary Figure 1

Comparison between SEQUEST-like and optimized Pytheas scoring function using 95 RNA spectra from the reference set. See **Reference RNA for training Pytheas scoring function** in **Methods** for further details on how the library of high-quality tandem spectra was compiled. (a)(c) Scatter plot of the S_p scores for target vs highest scoring competing decoy sequences (b)(d) Box and whisker plots represent distribution of the S_p scores for targets (blue) and decoys (red) across oligonucleotide sequence length using (b) SEQUEST-like or (d) optimized Pytheas scores. The whiskers represent the extremes, the center line is the median, and the box outlines the limits of the upper and lower quartiles. Source data are provided as a **Source Data** file.

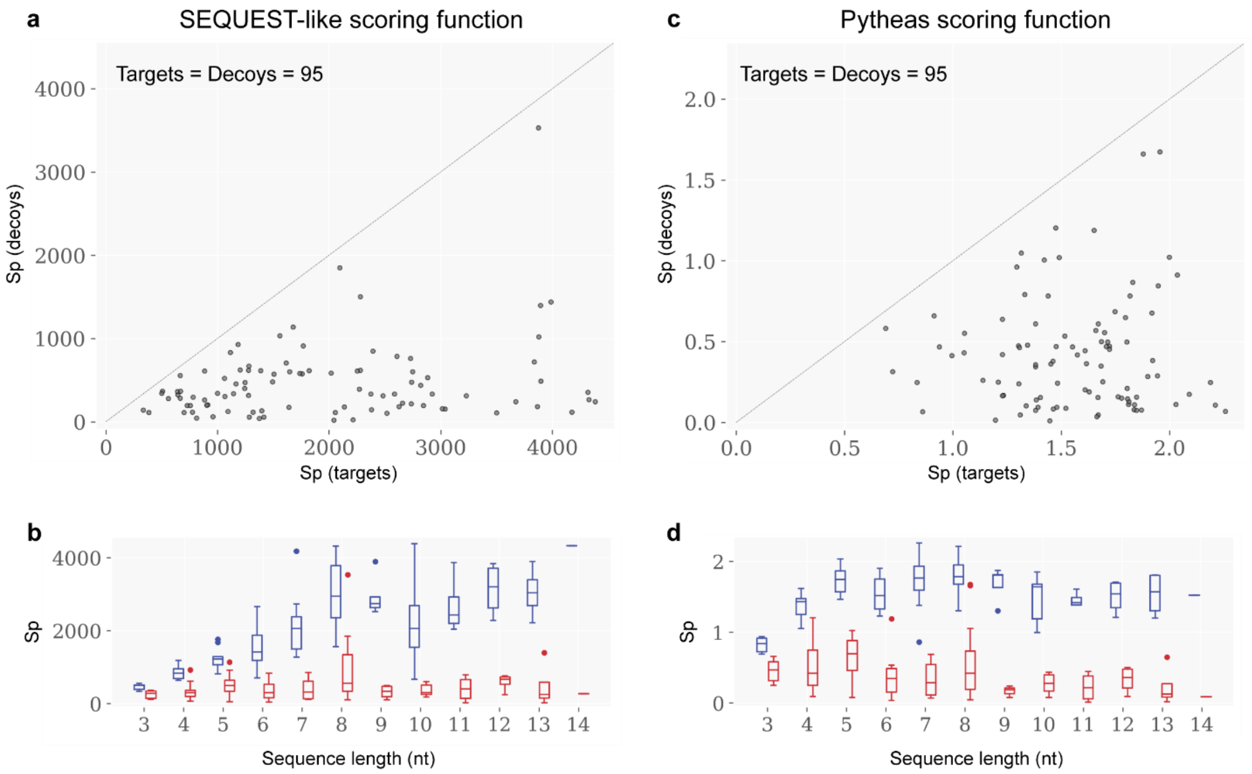

Supplementary Figure 2

Scatter plots of the Pytheas S_p scores for targets vs their highest scoring competing decoys clustered by sequence length. A total of 95 spectra present in the reference set were used here. Source data are provided as a **Source Data** file.

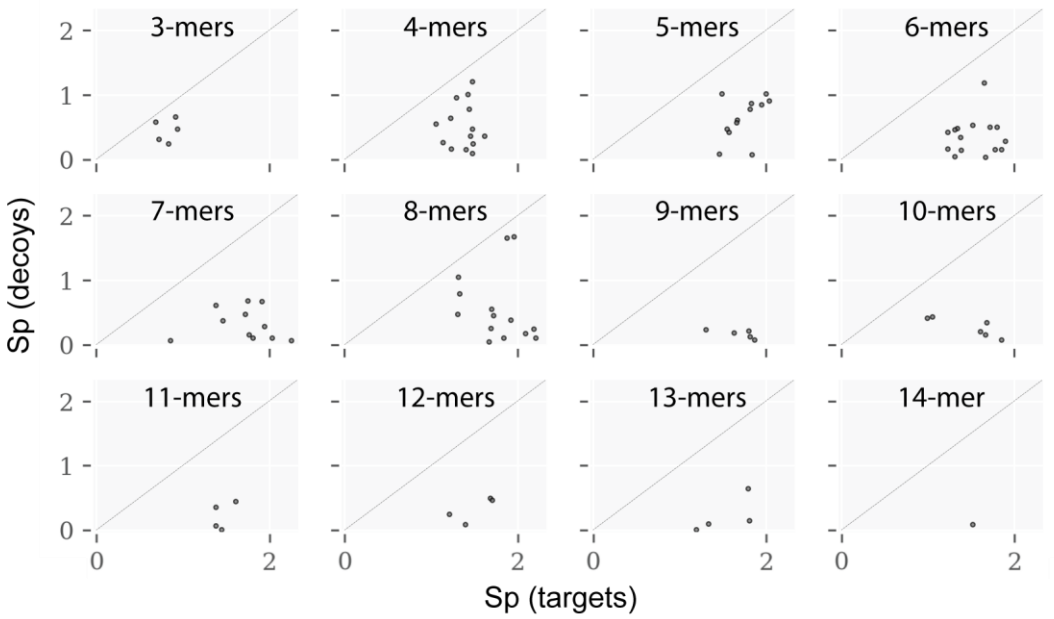

Supplementary Figure 3

Comparison of annotated tandem spectra from the *E. coli* 16S datasets acquired via Q-TOF, Synapt and Orbitrap MS instruments. Spectra were assigned to 1518-[m_2^6A][m_2^6A]CCUG-1523, where [m_2^6A] is N6,N6-dimethyladenosine. All are identified at the 1% FDR threshold in the respective dataset. For each spectrum, fragment ion matches are highlighted, color-coded based on their ion series (same color scheme as in **Fig. 2a**) and reported in the table below the spectrum. The tables show theoretically predicted m/z values. All the panels have been prepared via Pytheas visualization tools. Overall, good fragment ion coverage and occurrence of 5' and 3' read-through matches across multiple ion series is apparent independent of the MS instrument used.

a

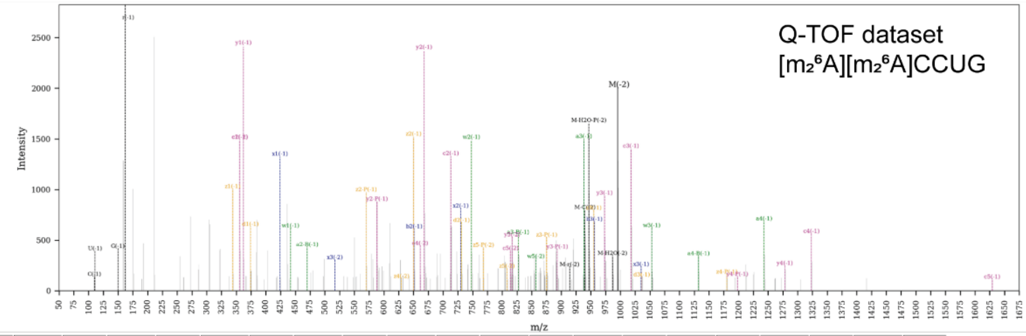

	a(-1)	a(-2)	a-B(-1)	a-B(-2)	b(-1)	b(-2)	c(-1)	c(-2)	d(-1)	d(-2)	w(-1)	w(-2)	x(-1)	x(-2)	y(-1)	y(-2)	y-P(-1)	y-P(-2)	z(-1)	z(-2)	z-P(-1)	z-P(-2)
r							356.076		374.0866													
r	633.1935	316.0928	470.1077		651.204	325.0981	713.1598	356.076	731.1704	365.0813	1715.2082	857.1002	1697.1976	848.0949	1635.2419	817.117	1555.2755	777.1339	1617.2313	808.1117	1537.265	768.1286
C	938.2348	468.6135	827.1915	413.0918	956.2453	477.6188	1018.2011	508.5966	1036.2117	517.6019	1358.1244	678.5583	1340.1138	669.553	1278.158	638.5751	1198.1917	598.5919	1260.1475	629.5698	1180.1812	589.5867
C	1243.2761	621.1341	1132.2328	565.6125	1261.2866	630.1394	1323.2424	661.1173	1341.253	670.1226	1053.0831	526.0376	1035.0725	517.0324	973.1168	486.0545	893.1504	446.0713	955.1062	477.0492	875.1399	437.066
U	1549.3014	774.1468	1437.2741	718.1331	1567.3119	783.1521	1629.2677	814.1299	1647.2783	823.1352	748.0418	373.517	730.0312	364.5117	668.0755	333.5338	588.1091		650.0649	324.5285	570.0986	
G											442.0165		424.0059		362.0502				344.0396			

C(-1)	G(-1)	r(-1)	U(-1)	M-P(-2)	M-P(-1)	M-C(-1)	M-C(-2)	M-G(-1)	M-G(-2)	M-r(-1)	M-r(-2)	M-U(-1)	M-U(-2)	M-H2O(-2)	M-P-C(-1)	M-P-C(-2)	M-P-G(-1)	M-P-G(-2)	M-P-r(-1)	M-P-r(-2)	M-P-U(-1)	M-P-U(-2)	M-H2O-P(-2)	M-H2O-P(-1)
110.0354	150.0416	162.078	111.0195	955.6758	1912.3594	1881.2824	940.1373	1841.2763	920.1342	1829.2399	914.116	1880.2984	939.6453	986.6536	1801.3161	900.1541	1761.3099	880.1511	1749.2736	874.1329	1800.3321	899.6621	946.6705	1894.3488

b

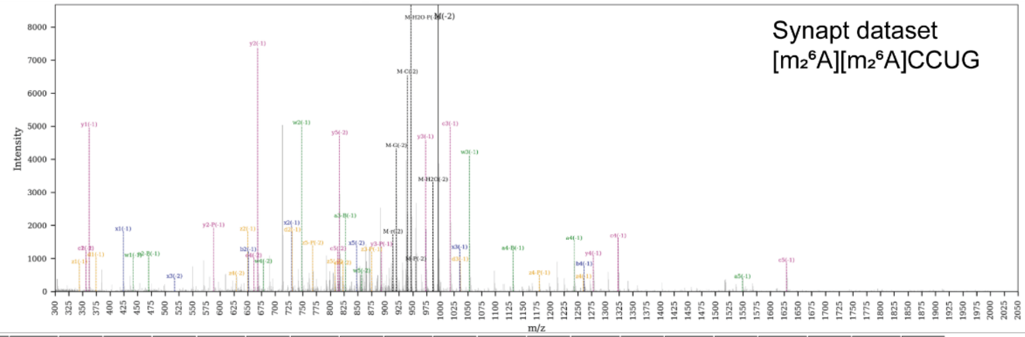

	a(-1)	a(-2)	a-B(-1)	a-B(-2)	b(-1)	b(-2)	c(-1)	c(-2)	d(-1)	d(-2)	w(-1)	w(-2)	x(-1)	x(-2)	y(-1)	y(-2)	y-P(-1)	y-P(-2)	z(-1)	z(-2)	z-P(-1)	z-P(-2)
r							356.076		374.0866													
r	633.1935	316.0928	470.1077		651.204	325.0981	713.1598	356.076	731.1704	365.0813	1715.2082	857.1002	1697.1976	848.0949	1635.2419	817.117	1555.2755	777.1339	1617.2313	808.1117	1537.265	768.1286
C	938.2348	468.6135	827.1915	413.0918	956.2453	477.6188	1018.2011	508.5966	1036.2117	517.6019	1358.1244	678.5583	1340.1138	669.553	1278.158	638.5751	1198.1917	598.5919	1260.1475	629.5698	1180.1812	589.5867
C	1243.2761	621.1341	1132.2328	565.6125	1261.2866	630.1394	1323.2424	661.1173	1341.253	670.1226	1053.0831	526.0376	1035.0725	517.0324	973.1168	486.0545	893.1504	446.0713	955.1062	477.0492	875.1399	437.066
U	1549.3014	774.1468	1437.2741	718.1331	1567.3119	783.1521	1629.2677	814.1299	1647.2783	823.1352	748.0418	373.517	730.0312	364.5117	668.0755	333.5338	588.1091		650.0649	324.5285	570.0986	
G											442.0165		424.0059		362.0502				344.0396			

C(-1)	G(-1)	r(-1)	U(-1)	M-P(-2)	M-P(-1)	M-C(-1)	M-C(-2)	M-G(-1)	M-G(-2)	M-r(-1)	M-r(-2)	M-U(-1)	M-U(-2)	M-H2O(-2)	M-P-C(-1)	M-P-C(-2)	M-P-G(-1)	M-P-G(-2)	M-P-r(-1)	M-P-r(-2)	M-P-U(-1)	M-P-U(-2)	M-H2O-P(-2)	M-H2O-P(-1)
110.0354	150.0416	162.078	111.0195	955.6758	1912.3594	1881.2824	940.1373	1841.2763	920.1342	1829.2399	914.116	1880.2984	939.6453	986.6536	1801.3161	900.1541	1761.3099	880.1511	1749.2736	874.1329	1800.3321	899.6621	946.6705	1894.3488

c

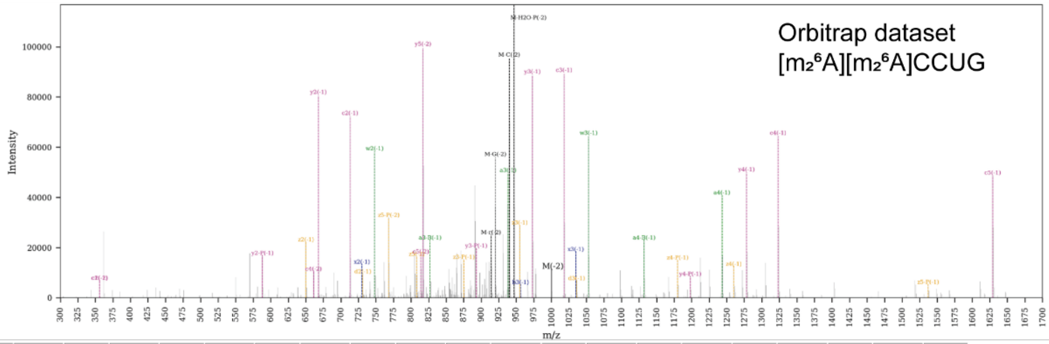

	a(-1)	a(-2)	a-B(-1)	a-B(-2)	b(-1)	b(-2)	c(-1)	c(-2)	d(-1)	d(-2)	w(-1)	w(-2)	x(-1)	x(-2)	y(-1)	y(-2)	y-P(-1)	y-P(-2)	z(-1)	z(-2)	z-P(-1)	z-P(-2)
r							356.076		374.0866													
r	633.1935	316.0928	470.1077		651.204	325.0981	713.1598	356.076	731.1704	365.0813	1715.2082	857.1002	1697.1976	848.0949	1635.2419	817.117	1555.2755	777.1339	1617.2313	808.1117	1537.265	768.1286
C	938.2348	468.6135	827.1915	413.0918	956.2453	477.6188	1018.2011	508.5966	1036.2117	517.6019	1358.1244	678.5583	1340.1138	669.553	1278.158	638.5751	1198.1917	598.5919	1260.1475	629.5698	1180.1812	589.5867
C	1243.2761	621.1341	1132.2328	565.6125	1261.2866	630.1394	1323.2424	661.1173	1341.253	670.1226	1053.0831	526.0376	1035.0725	517.0324	973.1168	486.0545	893.1504	446.0713	955.1062	477.0492	875.1399	437.066
U	1549.3014	774.1468	1437.2741	718.1331	1567.3119	783.1521	1629.2677	814.1299	1647.2783	823.1352	748.0418	373.517	730.0312	364.5117	668.0755	333.5338	588.1091		650.0649	324.5285	570.0986	
G											442.0165		424.0059		362.0502				344.0396			

C(-1)	G(-1)	r(-1)	U(-1)	M-P(-2)	M-P(-1)	M-C(-1)	M-C(-2)	M-G(-1)	M-G(-2)	M-r(-1)	M-r(-2)	M-U(-1)	M-U(-2)	M-H2O(-2)	M-P-C(-1)	M-P-C(-2)	M-P-G(-1)	M-P-G(-2)	M-P-r(-1)	M-P-r(-2)	M-P-U(-1)	M-P-U(-2)	M-H2O-P(-2)	M-H2O-P(-1)
110.0354	150.0416	162.078	111.0195	955.6758	1912.3594	1881.2824	940.1373	1841.2763	920.1342	1829.2399	914.116	1880.2984	939.6453	986.6537	1801.3161	900.1541	1761.3099	880.1511	1749.2736	874.1329	1800.3321	899.6621	946.6705	1894.3488

Supplementary Figure 4

Distributions of the S_p scores for top scoring targets and top scoring competing decoys across sequence length in three *E. coli* 16S datasets. (a) Q-TOF, (b) Synapt and (c) Orbitrap datasets. Data points for ^{14}N-and ^{15}N-labeled OSMs are reported, with a total number of targets/decoys shown in the upper right. Targets are in blue, and decoys are in red. 5% FDR threshold (dashed line) has been applied to targets, while their competing decoys are retained in the plots independent on their S_p value. Targets with at least one competing decoy are shown, and targets without decoys are excluded. Most of the 3-mer targets present in three datasets either have no competing decoys (due to decoy sampling problem for short sequences) or are below the S_p cutoff and are not shown. The box and whisker (bottom) plots in panels a-c demonstrate the distribution of S_p across length of T1 fragments identified within a single dataset. The whiskers represent the extremes, the center line is the median, and the box outlines the limits of the upper and lower quartiles**.** Source data are provided as a **Source Data** file.

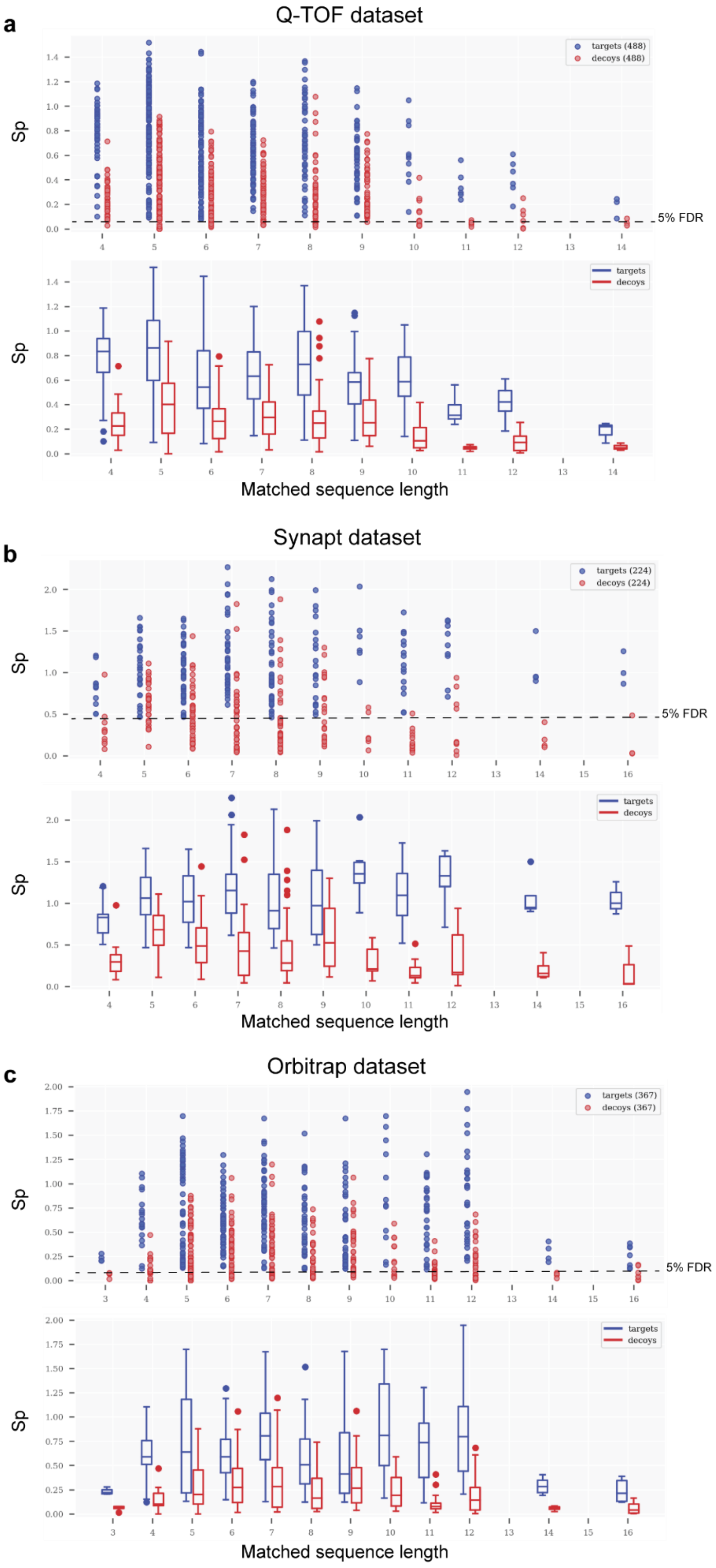
a
Q-TOF dataset
targets (488)
decoys (488)
5% FDR
targets
decoys
Sp
Matched sequence length
b
Synapt dataset
targets (224)
decoys (224)
5% FDR
targets
decoys
Sp
Matched sequence length
c
Orbitrap dataset
targets (367)
decoys (367)
5% FDR
targets
decoys
Sp
Matched sequence length

Supplementary Figure 5

Tandem spectrum assigned by Pytheas to a N7-methylguanosine [m^{7}G] containing sequence within the *E. coli* 16S. Fragment ion matches are highlighted, color-coded based on their ion series and reported in the table below the spectrum. Predicted *m/z* values are shown in the table. In contrast to other modified sequences within 16S dataset, 525-CC[m^{7}G]CG-529 spectrum displays incomplete fragment ion coverage, with no MS2 ions containing [m^{7}G] being matched. Two factors potentially contribute to the observed lack of [m^{7}G] sequence-defining ions: 1) +1 charge on the [m^{7}G] base 2) [m^{7}G] base losses. [m^{7}G] positive charge may result in net charge neutralization and lead to undetectable fragment ions. Nonetheless, ions containing [m^{7}G] base losses, such as c3-[m^{7}G] with predicted *m/z*=803.07 (pointed by the red arrow) may be included in the list of theoretically predicted to account for [m^{7}G]-specific peaks that currently remain unassigned by Pytheas.

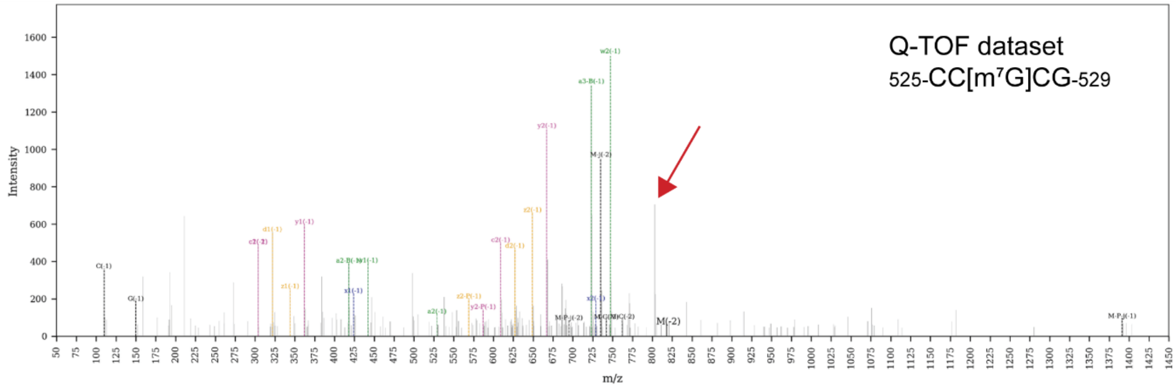

m/z

	a(-1)	a(-2)	a-B(-1)	a-B(-2)	b(-1)	b(-2)	c(-1)	c(-2)	d(-1)	d(-2)	w(-1)	w(-2)	x(-1)	x(-2)	y(-1)	y(-2)	y-P(-1)	y-P(-2)	z(-1)	z(-2)	z-P(-1)	z-P(-2)
C							304.0335		322.044													
C	529.1084		418.0652		547.119		609.0747	304.0335	627.0853	313.0387	1411.1622	705.0772	1393.1516	696.0719	1331.1958	665.094	1251.2295	625.1108	1313.1853	656.0887	1233.2189	616.1056
j	888.1715	443.5818	723.1064	361.0493	906.1821	452.5871	968.1378	483.565	986.1484	492.5703	1106.1209	552.5565	1088.1103	543.5512	1026.1545	512.5734	946.1882	472.5902	1008.144	503.5681	928.1777	463.5849
C	1193.2128	596.1025	1082.1695	540.5809	1211.2234	605.1078	1273.1791	636.0856	1291.1897	645.0909	747.0578	373.025	729.0472	364.0197	667.0915	333.0418	587.1251		649.0809	324.0365	569.1146	
G											442.0165		424.0059		362.0502				344.0396			

C(-1)	G(-1)	j(-1)	M-P(-2)	M-P(-1)	M-C(-1)	M-C(-2)	M-G(-1)	M-G(-2)	M-j(-1)	M-j(-2)	M-H2O(-2)	M-P-C(-1)	M-P-C(-2)	M-P-G(-1)	M-P-G(-2)	M-P-j(-1)	M-P-j(-2)	M-H2O-P(-2)	M-H2O-P(-1)
110.0354	150.0416	164.0572	777.6315	1556.2708	1525.1939	762.093	1485.1877	742.0899	1471.1721	735.0821	808.6094	1445.2275	722.1099	1405.2214	702.1068	1391.2057	695.099	768.6262	1538.2602

Supplementary Figure 6

List of 19 nucleoside modifications included in the *in silico* digest library for analysis of yeast tRNA dataset. Modifications full names, Pytheas one letter and extended notations are reported in the upper-left table. Chemical structures for PTxMs other than base/ribose methylations are shown with the additional moieties highlighted in red. tRNA pseudouridines were excluded from the search since they cannot be identified in absence of isotopic labeling or derivatization.

Name	ID	Extended ID
2'-O-methyladenosine	F	[Am]
2'-O-methylguanosine	J	[Gm]
2'-O-methylcytidine	K	[Cm]
2'-O-methyluridine	L	[Um]
methyl adenosine (base)	i	[mA]
methyl guanosine (base)	j	[mG]
methyl cytidine (base)	k	[mC]
methyl uridine (base)	l	[mU]
dimethylguanosine	g	[mmG]
inosine	I	[I]
dihydrouridine	D	[D]
5-methoxycarbonylmethyluridine	f	[mcm5U]
N6-threonylcarbamoyladenosine	o	[t6A]
N6-isopentenyladenosine	n	[i6A]
5-methoxycarbonylmethyl-2-thiouridine	m	[mcm5s2U]
N4-acetylcytidine	p	[ac4C]
5-carbamoylmethyluridine	q	[ncm5U]
2'-O-ribosyladenosine (phosphate)	t	[Ar(p)]
wybutosine	u	[yW]

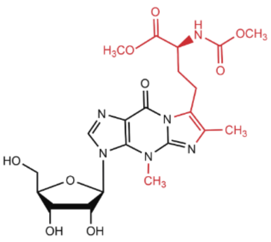

yW

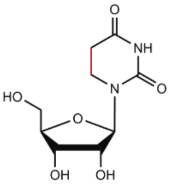

D

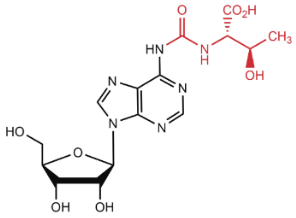

t6A

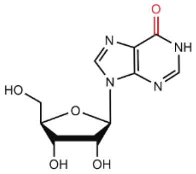

I

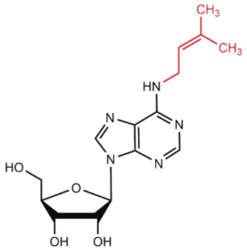

i6A

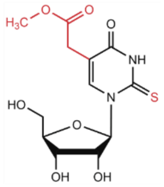

mcm5s2U

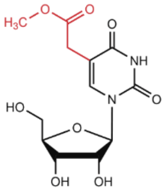

mcm5U

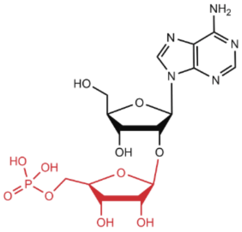

Ar(p)

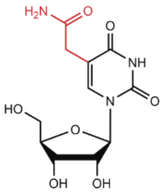

ncm5U

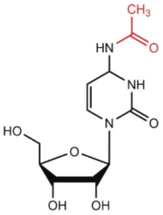

ac4C

Supplementary Figure 7

Tandem spectra identified by Pytheas and assigned to sequences containing chemically complex RNA modifications. (a) Spectrum assigned to 32-CU[mcm^{5}s^{2}U]UCACCG-40 from tRNA^{Glu}. (b) Spectrum assigned to 31-A[Cm]U[Gm]AA[yW]AU[mC]UG-42 from tRNA^{Phe}. For each spectrum, fragment ion matches are highlighted, color-coded based on their ion series and reported in the table below the spectra using predicted *m/z* values. Pytheas one letter and extended notations for modifications are the same as used in **Supplementary Fig. 6** and **Table 4**.

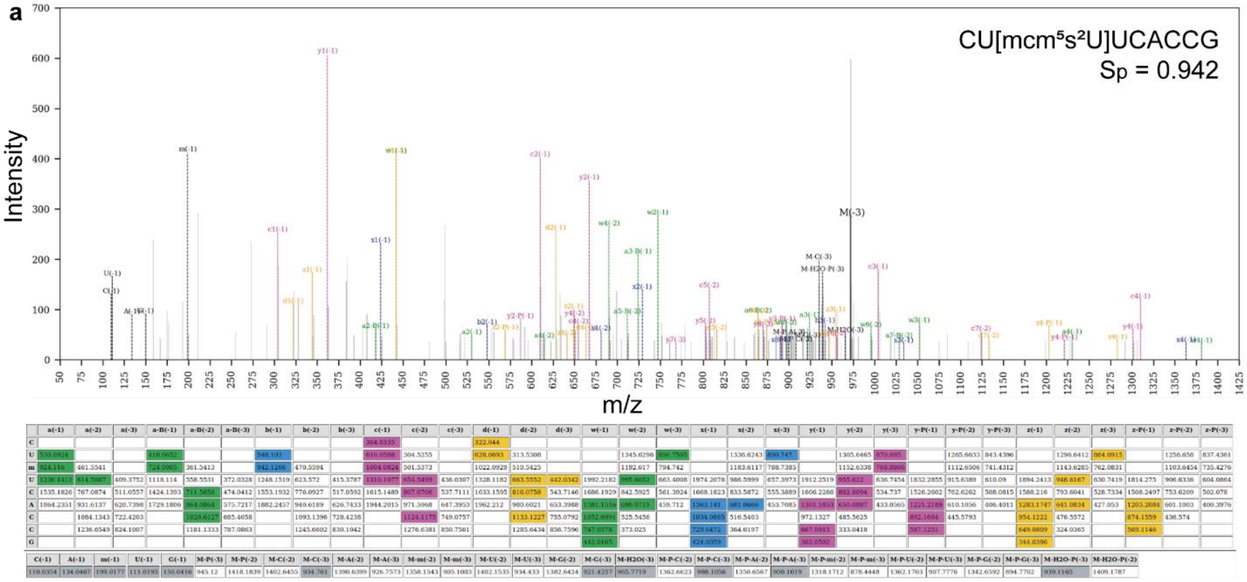

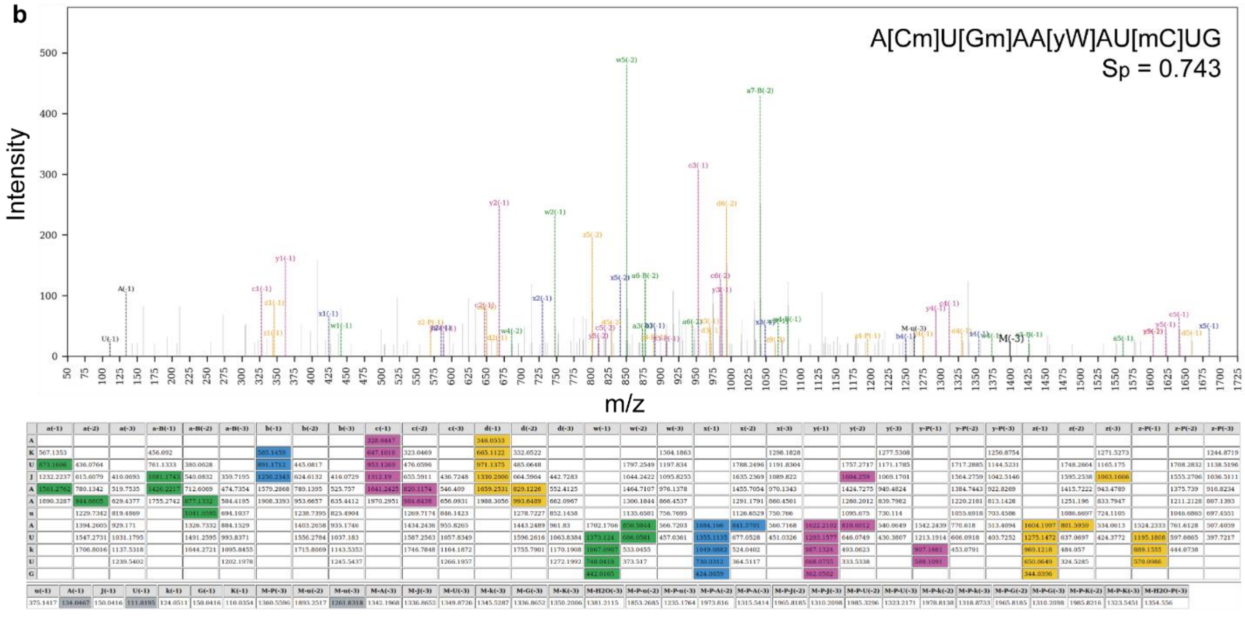

Supplementary Figure 8

Distributions of the S_p scores for top target sequence identifications in all-U and all-m$^1\Psi$ RNA datasets. Two 899 nt long mRNA constructs were *in vitro* transcribed from the linear DNA template coding for GFP with either all U or all m$^1\Psi$ 5'-triphosphates used in the transcription reaction. Each RNA sample was T1 digested and analyzed by Agilent Q-TOF. The search against GFP mRNA theoretical digest was performed by setting $\beta = 0.055$ and $\alpha = 2$, and by enabling precursor ion matching to *m/z* of M+1 and M-1 isotopologues. Targets with *S*p<0.275 (4% FDR in all-U dataset) were filtered out, and only sequences containing at least one U/ m$^1\Psi$ included in the analysis. Data points are organized by sequence length and '+' represent the median for each group of identifications. Total number of targets is show in the upper right. Source data are provided as a **Source Data** file. Overall, no *S*p biases between U- and m$^1\Psi$- targets were observed.

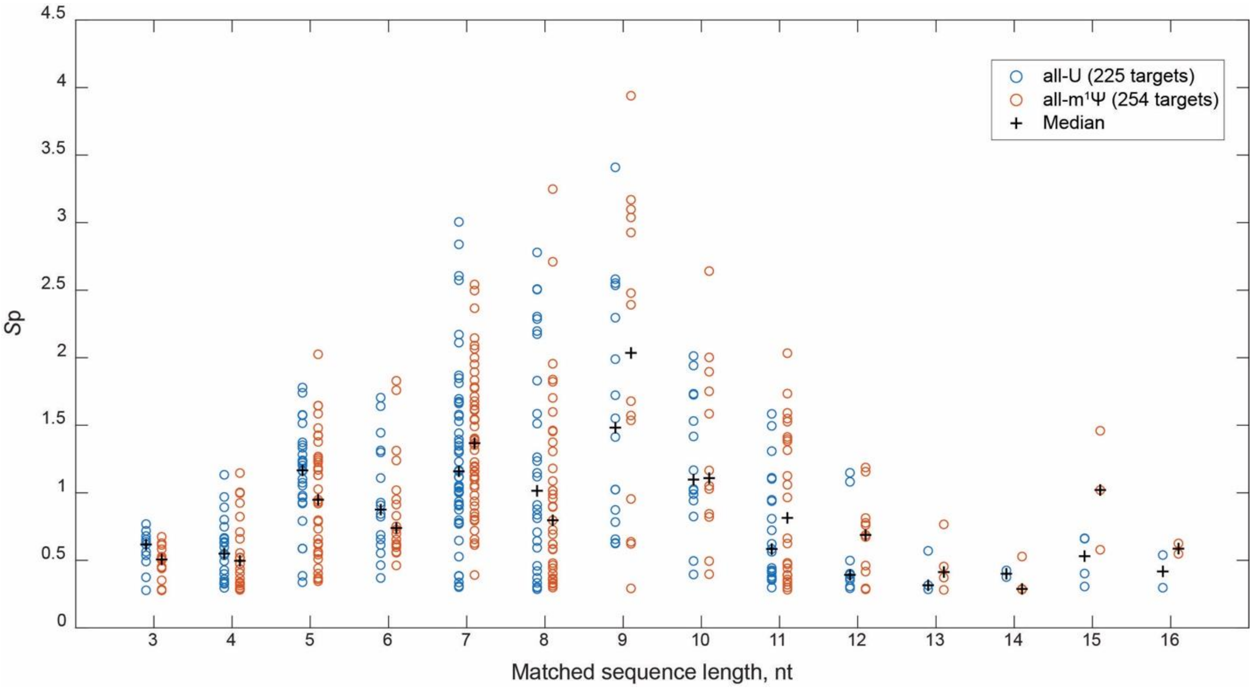

Supplementary Figure 9

Identification of RNA with 2',3'-cyclic phosphate (-cP) chemistry. An example of a tandem spectrum assigned to the nucleolytic fragment obtained following Cusativin cleavage. Fragment ion matches are highlighted, color-coded based on their ion series and reported in the table below the spectrum using predicted m/z values. Identification of RNA with -cP chemistry at the 3' terminus has been enabled in Pytheas, in addition to 3' -OH and -P (linear phosphate), to accommodate identification of nucleolytic fragments obtained at low RNase concentrations. Cusativin endonuclease has been recombinantly expressed and purified[2].

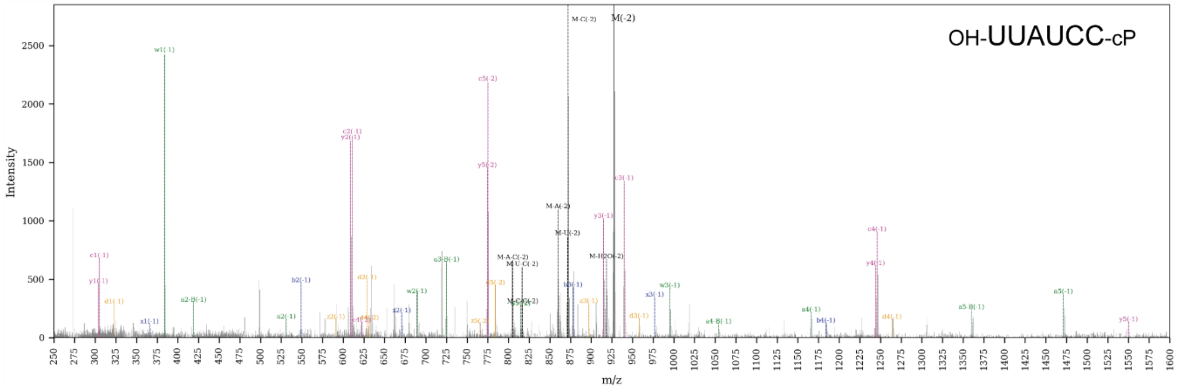

	a(-1)	a(-2)	a-B(-1)	a-B(-2)	b(-1)	b(-2)	c(-1)	c(-2)	d(-1)	d(-2)	w(-1)	w(-2)	x(-1)	x(-2)	y(-1)	y(-2)	z(-1)	z(-2)
U							305.0175		323.028									
U	531.0764		419.0492		549.087		611.0428		629.0533		1630.1442	814.5682	1612.1336	805.5629	1550.1779	774.585	1532.1673	765.5797
A	860.129	429.5606	725.0745	362.0333	878.1395	438.5659	940.0953	469.5437	958.1059	478.549	1324.1189	661.5555	1306.1083	652.5503	1244.1526	621.5724	1226.142	612.5671
U	1166.1543	582.5732	1054.127	526.5596	1184.1648	591.5785	1246.1206	622.5564	1264.1312	631.5617	995.0664	497.0293	977.0558	488.024	915.1001	457.0461	897.0895	448.0408
C	1471.1956	735.0939	1360.1523	679.5722	1489.2061	744.0991	1551.1619	775.077	1569.1725	784.0823	689.0411		671.0305		609.0747		591.0642	
C											383.9998		365.9892		304.0335			

A(-1)	U(-1)	C(-1)	M-P(-1)	M-A(-1)	M-A(-2)	M-U(-1)	M-U(-2)	M-C(-1)	M-C(-2)	M-H2O(-2)	M-P-C(-1)	M-A-C(-1)	M-A-C(-2)	M-U-C(-1)	M-U-C(-2)	M-C-C(-1)	M-C-C(-2)
134.0467	111.0195	110.0354	1776.2368	1721.1487	860.0704	1744.1759	871.584	1745.1599	872.076	918.5924	1665.1936	1611.1132	805.0527	1634.1405	816.5663	1635.1245	817.0583

Supplementary Figure 10

Support of positive ionization mode. Analysis of RNA MS/MS data acquired in positive ionization mode is implemented in Pytheas by selecting "positive" ion mode option at the *in silico* digestion step. As an example, we acquired MS/MS data of an 8-mer synthetic oligonucleotide with Agilent Q-TOF. The data were matched against the theoretical library obtained using an extensive set of MS2 charge states and fragmentation series. This was done due to the limited availability of RNA data at positive ionization. Fragment ion matches are highlighted, color-coded based on their ion series and reported in the table below. The table shows theoretically predicted m/z values. Usually, spectra acquired via negative ionization consistently display MS2 ions from 9 fragmentation series (shown in the table header), however positive mode spectrum above is mainly represented by c/y (red) and a/a-B/w (green) fragments.

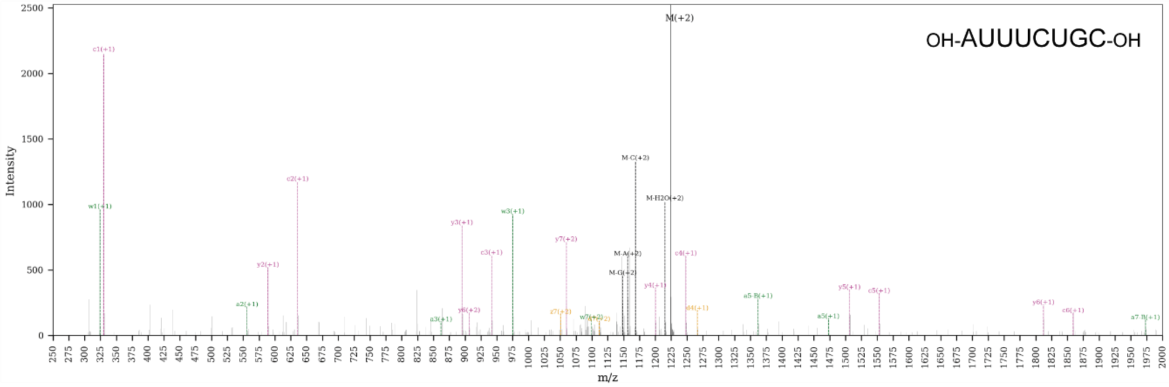

	a(+1)	a(+2)	a-B(+1)	a-B(+2)	b(+1)	b(+2)	c(+1)	c(+2)	d(+1)	d(+2)	w(+1)	w(+2)	x(+1)	x(+2)	y(+1)	y(+2)	z(+1)	z(+2)
A							330.0603		348.0709									
U	556.1193		444.092		574.1299		636.0856		654.0962			1099.6287		1090.6234		1059.6456		1050.6403
U	862.1446	431.5762	750.1173	375.5626	880.1552	440.5815	942.111	471.5594	960.1215	480.5647	1892.2243	946.6161	1874.2137	937.6108	1812.258	906.6329	1794.2474	897.6276
U	1168.1699	584.5889	1056.1426	528.5752	1186.1805	593.5942	1248.1363	624.572	1266.1468	633.5773	1586.199	793.6034	1568.1884	784.5981	1506.2327	753.6202	1488.2221	744.615
C	1473.2112	737.1095	1362.1679	681.5879	1491.2218	746.1148	1553.1775	777.0927	1571.1881	786.098	1280.1737	640.5908	1262.1631	631.5855	1200.2074	600.6076	1182.1968	591.6023
U	1779.2365	890.1222	1667.2092	834.1085	1797.2471	899.1274	1859.2028	930.1053	1877.2134	939.1106	975.1324	488.0701	957.1218	479.0648	895.1661	448.087	877.1555	439.0817
G		1062.6459	1973.2345	987.1212		1071.6512		1102.6291		1111.6343	669.1071		651.0965		589.1408		571.1302	
C											324.0597		306.0491					

A(+1)	G(+1)	C(+1)	U(+1)	M-A(+2)	M-G(+2)	M-C(+2)	M-U(+2)	M-H2O(+2)
136.0623	152.0572	112.0511	113.0351	1156.6446	1148.6471	1168.6502	1168.1582	1215.1665

Supplementary Figure 11

Support of dissociation methods other than Collision Induced Dissociation (CID). Although Pytheas development involved the exclusive usage of MS/MS data obtained via CID, the flexibility of its routine allows the user to analyze MS/MS data obtained with different dissociation methods. As a proof of principle, we performed Pytheas search on a rRNA dataset acquired with Higher-energy C-trap dissociation (HCD) by the NASE developers[3] and freely available online (web link: https://www.ebi.ac.uk/pride/archive/projects/PXD016323). The dataset was matched against a theoretical T1 digest of human 18S and 28S rRNA. An **e**xample of an HCD spectrum assigned to OH-AUCCCG-P from human 28S RNA is shown. Fragment ion matches are highlighted, color-coded based on their ion series and reported in the table below. The table shows theoretically predicted m/z values. The assigned spectrum demonstrates good coverage for all eleven fragment ion series, with the presence of consecutive matched ions and 3’ and 5’ sequence read-through.

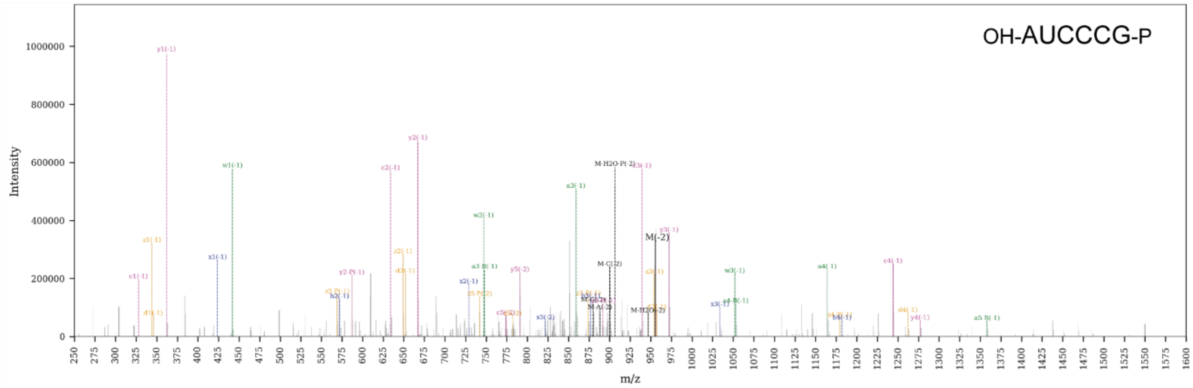

	a(-1)	a(-2)	a-B(-1)	a-B(-2)	b(-1)	b(-2)	c(-1)	c(-2)	d(-1)	d(-2)	w(-1)	w(-2)	x(-1)	x(-2)	y(-1)	y(-2)	y-P(-1)	y-P(-2)	z(-1)	z(-2)	z-P(-1)	z-P(-2)
A							328.0447		346.0553													
U	554.1037		442.0764		572.1142		634.07		652.0806		1663.1657	831.0789	1645.1551	822.0736	1583.1993	791.0958	1503.233	751.1126	1565.1888	782.0905	1485.2224	742.1073
C	859.145	429.0686	748.1017	373.5469	877.1555	438.0738	939.1113	469.0517	957.1218	478.057	1357.1404	678.0663	1339.1298	669.061	1277.174	638.0831	1197.2077	598.0999	1259.1635	629.0778	1179.1971	589.0947
C	1164.1862	581.5892	1053.143	526.0676	1182.1968	590.5945	1244.1526	621.5724	1262.1631	630.5777	1052.0991	525.5456	1034.0885	516.5403	972.1327	485.5625	892.1664	445.5793	954.1222	476.5572	874.1559	436.574
C	1469.2275	734.1099	1358.1843	678.5882	1487.2381	743.1151	1549.1939	774.093	1567.2044	783.0983	747.0578		729.0472		667.0915		587.1251		649.0809		569.1146	
G											442.0165		424.0059		362.0502				344.0396			

A(-1)	G(-1)	U(-1)	C(-1)	M-P(-2)	M-P(-1)	M-A(-1)	M-A(-2)	M-G(-1)	M-G(-2)	M-U(-1)	M-U(-2)	M-C(-1)	M-C(-2)	M-H2O(-2)	M-P-A(-1)	M-P-A(-2)	M-P-G(-1)	M-P-G(-2)	M-P-U(-1)	M-P-U(-2)	M-P-C(-1)	M-P-C(-2)	M-H2O-P(-2)	M-H2O-P(-1)
134.0467	150.0416	111.0195	110.0354	915.6389	1832.2855	1777.1974	888.0948	1761.2024	880.0973	1800.2246	899.6084	1801.2086	900.1004	946.6167	1697.231	848.1116	1681.2361	840.1141	1720.2582	859.6252	1721.2423	860.1172	906.6336	1814.275

Supplementary Figure 12

An example of the output from Pytheas spectra matching and scoring subroutine. Database search was performed against the *in silico* digest library generated for the analysis of the 16S datasets presented in this study. Precursor ion with experimental *m/z* = 543.398132 and RT = 35.89 min present in the Q-TOF .mgf file is matched against six candidate sequences from the theoretical digest. Each of six OSMs is assigned S_p score, rank, and ΔS_p score.

For the top OSM (rank 1): TH_MATCH = 543.39672307 refers to the theoretically predicted precursor mass, and 3 ppm is MS1 mass identification error. #MS2 = 27 is the number of matched sequence-defining MS2 ions; light refers to the isotope composition of RNA; 5 is the sequence length, and -3 is the assigned charge. AUUAG is the identified sequence, and OH P refers to the 5'-OH and 3'-P termini. 16S.ecoli, 787,791; 16S.ecoli, 243,247 show molecule ID and sequence location. AUUAG is the target sequence that is found at positions 787-791 and 243-247 in the *E. coli* 16S.

Rank 2 and 3 OSMs are represented by the competing decoy sequences present in the 16S digest library used for matching.

```
PRECURSOR_ION=543.398132
543.398132 RT=35.89 TH_MATCH=543.39672307 3ppm Sp=0.358 dSp=0.0 rank=1 #MS2=27 light 5 -3 AUUAG - OH P 16S.ecoli,787,791;16S.ecoli,243,247 [MS2 ions]
543.398132 RT=35.89 TH_MATCH=543.39672307 3ppm Sp=0.215 dSp=0.4 rank=2 #MS2=24 light 5 -3 UAUAG - OH P decoy [MS2 ions]
543.398132 RT=35.89 TH_MATCH=543.39672307 3ppm Sp=0.121 dSp=0.66 rank=3 #MS2=19 light 5 -3 AUAUG - OH P decoy [MS2 ions]
543.398132 RT=35.89 TH_MATCH=543.39672307 3ppm Sp=0.109 dSp=0.7 rank=4 #MS2=18 light 5 -3 UUAAG - OH P 16S.ecoli,593,597;16S.ecoli,870,874 [MS2 ions]
543.398132 RT=35.89 TH_MATCH=543.39672307 3ppm Sp=0.076 dSp=0.79 rank=5 #MS2=15 light 5 -3 AAUUG - OH P 16S.ecoli,918,922 [MS2 ions]
543.398132 RT=35.89 TH_MATCH=543.39672307 3ppm Sp=0.057 dSp=0.84 rank=6 #MS2=16 light 5 -3 UAAUG - OH P 16S.ecoli,118,122 [MS2 ions]
```

Supplementary Figure 13

An example of the graphical output from the Pytheas sequence mapping. Nucleolytic oligonucleotides identified by Pytheas in T1 digested SARS-CoV-2 spike protein mRNA sample are mapped back on the 3995 nt mRNA sequence (**Supplementary Fig. 15**). Rank 1 targets have been selected for mapping by applying (a) 3% and (b) 1% FDR cutoffs. mRNA sequence from .fasta input file is shown with nucleotide numbering on top and identified nucleolytic oligos are represented with colored bars. One bar represents one sequence identification, and multiple bars stacked upon one another represent multiple identifications of the same sequence. Modified nucleosides are shown as black squares, and 1 stands for N1-methylpseudouridine. The bars are colored based on S_p scores as follows. 100-66% of the maximum S_p value are colored red, 66-33% are orange and 33-0% are yellow. Compared to 3% FDR cutoff (a), 1% FDR (b) noticeably reduces number of low-confidence identifications (i.e., fewer yellow bars after filtering).

a

b

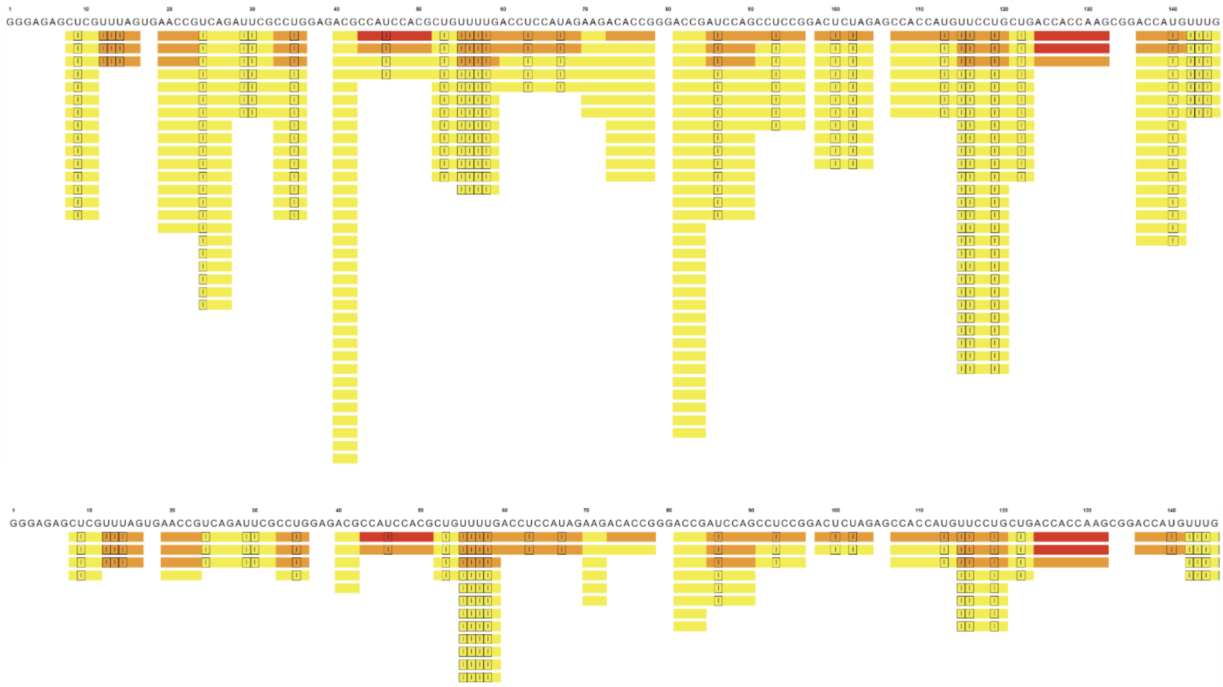

Supplementary Figure 14

Quality control of the SARS-CoV-2 spike protein mRNA produced using in-vitro transcription. ^{14}N-GTP and ^{15}N-GTP labeled 3995 nt long mRNA have been analyzed using TAE-based 1% agarose gel alongside 1kb DNA ladder (NEB) in the control lane. mRNAs are fully substituted with N1-methylpseudouridine. Gel has been stained with EtBr.

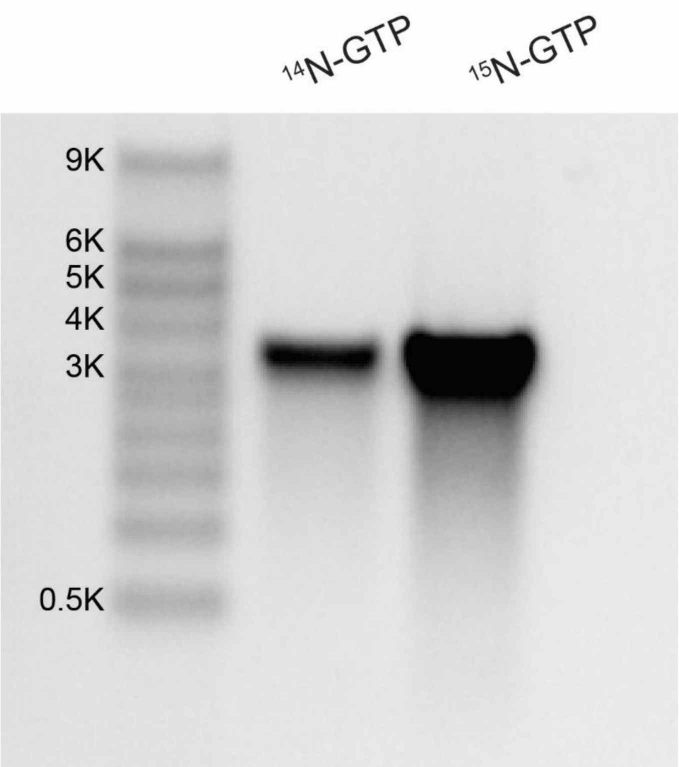

Supplementary Figure 15

Sequence of 3995 nt RNA analyzed in this work. Region corresponding to SARS-CoV-2 spike protein is highlighted in navy.

```
GGGAGAGCUC GUUUAGUGAA CCGUCAGAUU CGCCUGGAGA CGCCAUCCAC GCUGUUUUGA CCUCCAUAGA AGACACCGGG ACCGAUCCAG
CCUCCGGACU CUAGAGCCAC CAUGUUCCUG CUGACCACCA AGCGGACCAU GUUUGUCUUC CUGGUCCUGC UGCCCCUGGU CUCUUCACAG
UGCGUCAAUC UGACUACACG AACUCAGCUG CCCCCUGCUU AUACCAAUUC CUUCACCAGG GGCGUGUACU AUCCAGACAA GGUGUUUCGC
AGCUCCGUGC UGCACUCUAC ACAGGAUCUG UUUCUGCCCU UCUUUAGCAA CGUGACCUGG UUCCACGCCA UCCACGUGAG CGGCACCAAU
GGCACAAAGA GGUUCGACAA UCCCGUGCUG CCUUUUAACG AUGGCGUGUA CUUCGCCAGC ACCGAGAAGU CCAACAUCAU CCGCGGCUGG
AUCUUUGGCA CCACACUGGA CUCUAAGACA CAGAGCCUGC UGAUCGUGAA CAAUGCCACC AACGUGGUCA UCAAGGUGUG CGAGUUCCAG
UUUUGUAAUG AUCCUUUCCU GGGCGUGUAC UAUCACAAGA ACAAUAAGUC CUGGAUGGAG UCUGAGUUUA GGGUGUAUUC UAGCGCCAAC
AAUUGCACAU UUGAGUACGU GUCCCAGCCA UUCCUGAUGG ACCUGGAGGG CAAGCAGGGC AAUUUCAAGA ACCUGCGGGA GUUCGUGUUU
AAGAAUAUCG AUGGCUACUU CAAGAUCUAC UCCAAGCACA CCCCCAUCAA CCUGGUGCGG GACCUGCCAC AGGGCUUCUC UGCCCUGGAG
CCACUGGUGG AUCUGCCCAU CGGCAUCAAC AUCACCAGGU UUCAGACACU GCUGGCCCUG CACCGCAGCU ACCUGACACC UGGCGACUCC
UCUAGCGGAU GGACCGCAGG AGCUGCCGCC UACUAUGUGG GCUAUCUGCA GCCAAGGACC UUCCUGCUGA AGUACAACGA GAAUGGCACC
AUCACAGACG CCGUGGAUUG CGCCCUGGAU CCACUGAGCG AGACAAAGUG UACACUGAAG UCCUUUACCG UGGAGAAGGG CAUCUAUCAG
ACAUCCAAUU UCCGGGUGCA GCCCACCGAG UCUAUCGUGA GAUUUCCCAA UAUCACAAAC CUGUGCCCUU UUGGCGAGGU GUUCAACGCA
ACCAGGUUCG CAAGCGUGUA CGCAUGGAAU AGGAAGCGCA UCUCUAACUG CGUGGCCGAC UAUAGCGUGC UGUACAACUC CGCCUCUUUC
AGCACCUUUA AGUGCUAUGG CGUGAGCCCU ACAAAGCUGA AUGACCUGUG CUUUACCAAC GUGUACGCCG AUUCCUUCGU GAUCAGGGGC
GACGAGGUGC GCCAGAUCGC ACCAGGACAG ACAGGCAAGA UCGCAGACUA CAAUUAUAAG CUGCCUGACG AUUUCACCGG CUGCGUGAUC
GCCUGGAACU CCAACAAUCU GGAUUCUAAA GUGGGCGGCA ACUACAAUUA UCUGUACCGG CUGUUUAGAA AGUCUAAUCU GAAGCCUUUC
GAGCGGGACA UCUCUACAGA GAUCUACCAG GCCGGCAGCA CCCCAUGCAA UGGCGUGGAG GGCUUUAACU GUUAUUUCCC UCUGCAGAGC
UACGGCUUCC AGCCAACAAA CGGCGUGGGC UAUCAGCCCU ACAGAGUGGU GGUGCUGUCU UUUGAGCUGC UGCACGCACC UGCAACAGUG
UGCGGACCAA AGAAGAGCAC CAAUCUGGUG AAGAACAAGU GCGUGAACUU CAACUUCAAC GGCCUGACCG GAACAGGCGU GCUGACCGAG
UCCAACAAGA AGUUCCUGCC CUUUCAGCAG UUCGGCCGGG ACAUCGCAGA UACCACAGAC GCCGUGCGGG ACCCCCAGAC CCUGGAGAUC
CUGGACAUCA CACCAUGCAG CUUCGGCGGC GUGUCCGUGA UCACACCUGG CACCAAUACA AGCAACCAGG UGGCCGUGCU GUAUCAGGAC
GUGAAUUGUA CCGAGGUGCC AGUGGCAAUC CACGCAGAUC AGCUGACCCC UACAUGGAGG GUGUACAGCA CCGGCUCCAA CGUGUUCCAG
ACAAGGGCAG GAUGCCUGAU CGGAGCAGAG CACGUGAACA AUUCCUAUGA GUGCGACAUC CCCAUCGGCG CCGGCAUCUG UGCCUCUUAC
CAGACCCAGA CAAACAGCCC UGGCAGCGCA AGCUCCGUGG CAUCCCAGUC UAUCAUCGCC UAUACCAUGU CUCUGGGCGC CGAGAAUAGC
GUGGCCUACU CUAACAAUAG CAUCGCCAUC CCUACCAACU UCACAAUCUC CGUGACCACA GAGAUCCUGC CAGUGUCUAU GACCAAGACA
AGCGUGGACU GCACAAUGUA UAUCUGUGGC GAUAGCACCG AGUGCUCCAA CCUGCUGCUG CAGUACGGCA GCUUUUGUAC CCAGCUGAAU
AGAGCCCUGA CAGGCAUCGC CGUGGAGCAG GAUAAGAACA CACAGGAGGU GUUCGCCCAG GUGAAGCAGA UCUACAAGAC CCCCCCUAUC
AAGGACUUUG GCGGCUUCAA CUUCAGCCAG AUCCUGCCUG AUCCAUCCAA GCCAUCUAAG AGGAGCUUUA UCGAGGACCU GCUGUUCAAC
AAGGUGACCC UGGCCGAUGC CGGCUUCAUC AAGCAGUAUG GCGAUUGCCU GGGCGACAUC GCAGCACGCG ACCUGAUCUG UGCCCAGAAG
UUUAAUGGCC UGACCGUGCU GCCACCCCUG CUGACAGAUG AGAUGAUCGC ACAGUACACA AGCGCCCUGC UGGCAGGAAC CAUCACAUCC
GGAUGGACCU UCGGCGCAGG AGCCGCCCUG CAGAUCCCCU UUGCCAUGCA GAUGGCCUAU AGAUUCAACG GCAUCGGCGU GACCCAGAAU
GUGCUGUACG AGAACCAGAA GCUGAUCGCC AAUCAGUUUA ACUCCGCCAU CGGCAAGAUC CAGGACUCCC UGUCCUCUAC AGCCUCUGCC
CUGGGCAAGC UGCAGGAUGU GGUGAAUCAG AACGCCCAGG CCCUGAAUAC CCUGGUGAAG CAGCUGAGCU CCAACUUCGG CGCCAUCUCU
AGCGUGCUGA AUGACAUCCU GAGCCGGCUG GACCCACCGG AGGCAGAGGU GCAGAUCGAC CGGCUGAUCA CAGGCAGACU GCAGUCCCUG
CAGACCUAUG UGACACAGCA GCUGAUCAGG GCAGCAGAGA UCAGGGCAUC UGCCAAUCUG GCCGCCACCA AGAUGUCCGA GUGCGUGCUG
GGCCAGUCUA AGAGAGUGGA CUUUUGUGGC AAGGGCUAUC ACCUGAUGUC UUUCCCACAG AGCGCCCCUC ACGGAGUGGU GUUUCUGCAC
GUGACCUACG UGCCAGCCCA GGAGAAGAAC UUCACCACAG CACCAGCAAU CUGCCACGAU GGCAAGGCAC ACUUUCCAAG GGAGGGCGUG
UUCGUGAGCA ACGGAACCCA CUGGUUUGUG ACACAGAGAA AUUUCUACGA GCCUCAGAUC AUCACCACAG ACAAUACCUU CGUGAGCGGC
AACUGUGACG UGGUCAUCGG CAUCGUGAAC AAUACCGUGU AUGAUCCACU GCAGCCCGAG CUGGACAGCU UUAAGGAGGA GCUGGAUAAG
UACUUCAAGA AUCACACCUC CCCCGACGUG GAUCUGGGCG ACAUCUCCGG CAUCAAUGCC UCUGUGGUGA ACAUCCAGAA GGAGAUCGAC
AGGCUGAACG AGGUGGCCAA GAAUCUGAAC GAGUCUCUGA UCGAUCUGCA GGAGCUGGGC AAGUAUGAGC AGUACAUCAA GUGGCCCUGG
UACAUCUGGC UGGGCUUUAU CGCCGGCCUG AUCGCCAUCG UGAUGGUGAC CAUCAUGCUG UGCUGCAUGA CCAGCUGCUG CUCCUGCCUG
AAGGGCUGCU GCAGCUGCGG GAGCUGCUGC AAGUUCGACG AGGACGACAG CGAGCCCGUG CUGAAAGGCG UGAAGCUGCA CUACACCUGA
GCGGCCGCAA GGGUUCGAUC CCUACCGGUU AGUAA
```

Supplementary Figure 16

Optimization of RNA collision energies for Agilent Q-TOF 6520. To avoid over- or under-fragmentation, collision energies (CEs) were derived empirically using a set of 23 synthetic RNA oligonucleotides (IDT, Sigma, Dharmacon) that are 3-15 nt long, with either 3'-OH or 3'-P termini. Each oligo at 15-100 μM concentration (15 mM ammonium acetate, pH = 8.5 in 10% acetonitrile) was delivered to the ESI source by direct infusion. MS2 spectra were collected for 2-3 most abundant precursor charge states, at ten different CEs in the 6-50 V range with a 2 V step interval. An average of ~10 MS2 scans for each CE value was centroided, converted to **.**mgf, and scored using $S_{CE} = \sum I_{abs} * n/L$ metric (where n is the number of matched and L is the number of predicted MS2 ions, and $\sum I_{abs}$ is the sum of absolute peak intensities for n matches). For each precursor *m/z*, an interval of CEs within 15% of max S_{CE} was obtained. Vertical bars on the plots show the interval of CEs, and point markers show the average CE value within each interval. The resulting (*m/z*, *CE*) pairs were used to derive a linear fit equation $CE = k * (m/z) + b$, for three different precursor charge states (ch=2, 3, >3). Parameters k and b were used to set up the method in MassHunter LC/MS Acquisition B.06.01. *r* is a coefficient of regression. Source data are provided as a **Source Data** file.

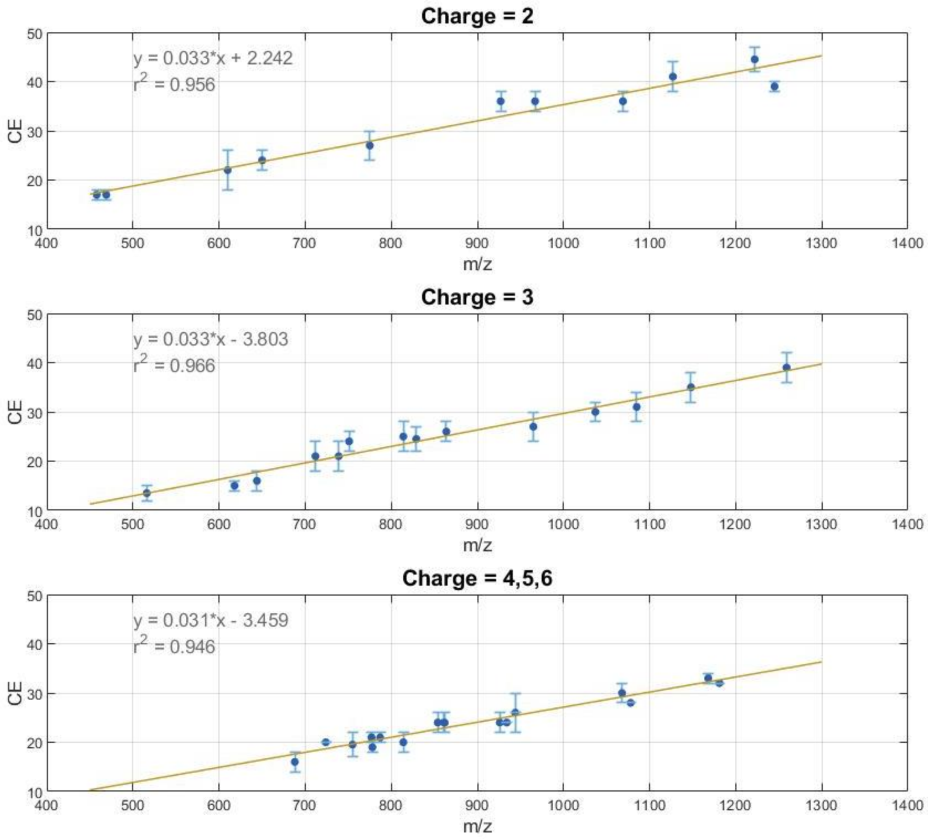

Supplementary Figure 17

Distributions of the mass offsets for precursor and fragment ions within *E. coli* 16S datasets acquired via three different MS instruments. Matching offsets for Agilent Q-TOF (a-b), Waters Synapt (c-d), and Thermo Scientific Orbitrap Fusion Lumos (e-f). Plots were obtained from preliminary analysis of the 16S data used to narrow down mass tolerance parameters for SeqX consolidation and final matching. The offset values included in the histograms were computed for ^{14}N-labeled rank 1 targets ONLY, following matching without SeqX. Comparison between the standard deviation values (σ, reported in the green boxes) identified Agilent Q-TOF as the least accurate instrument. For simplicity of the downstream analysis, Q-TOF 2σ values were used to obtain MS1 (16 ppm) and MS2 (40 ppm) tolerance for SeqX consolidation (applied during *in silico* digestion that included BOTH ^{14}N- and ^{15}N-labeled species) and for the final database matching. In the green boxes, μ is the mean, M is the median, and σ is a standard deviation. The figures were obtained directly from Pytheas statistical analysis tools. Total number of mass offsets used is indicted in the upper right. Source data are provided as a **Source Data** file.

a

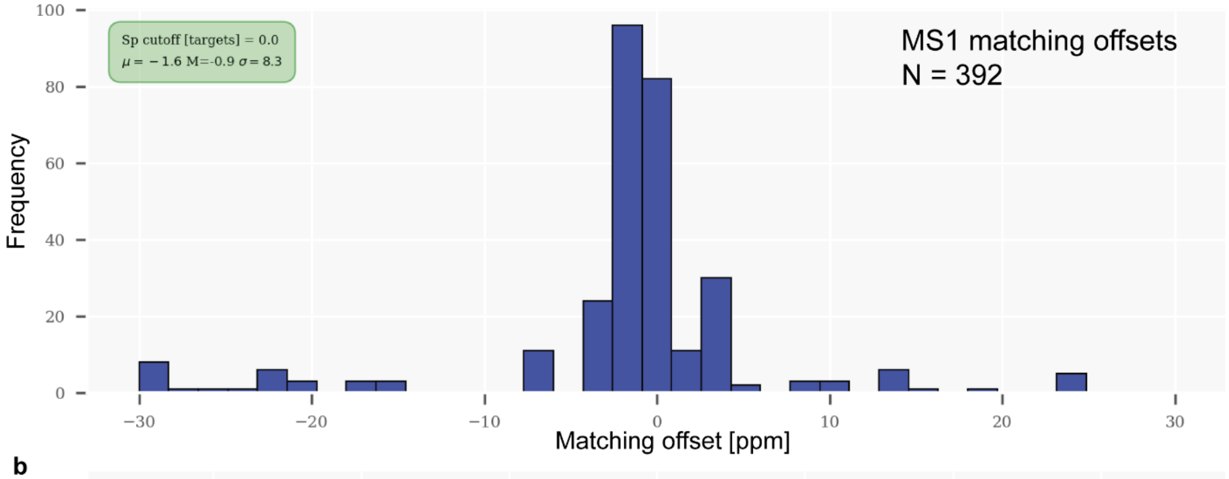

b

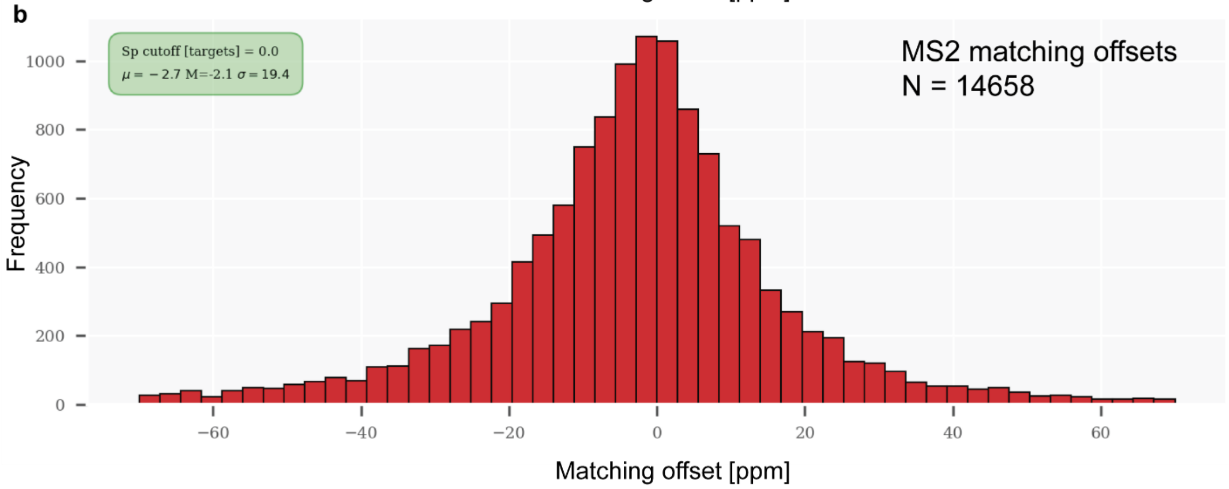

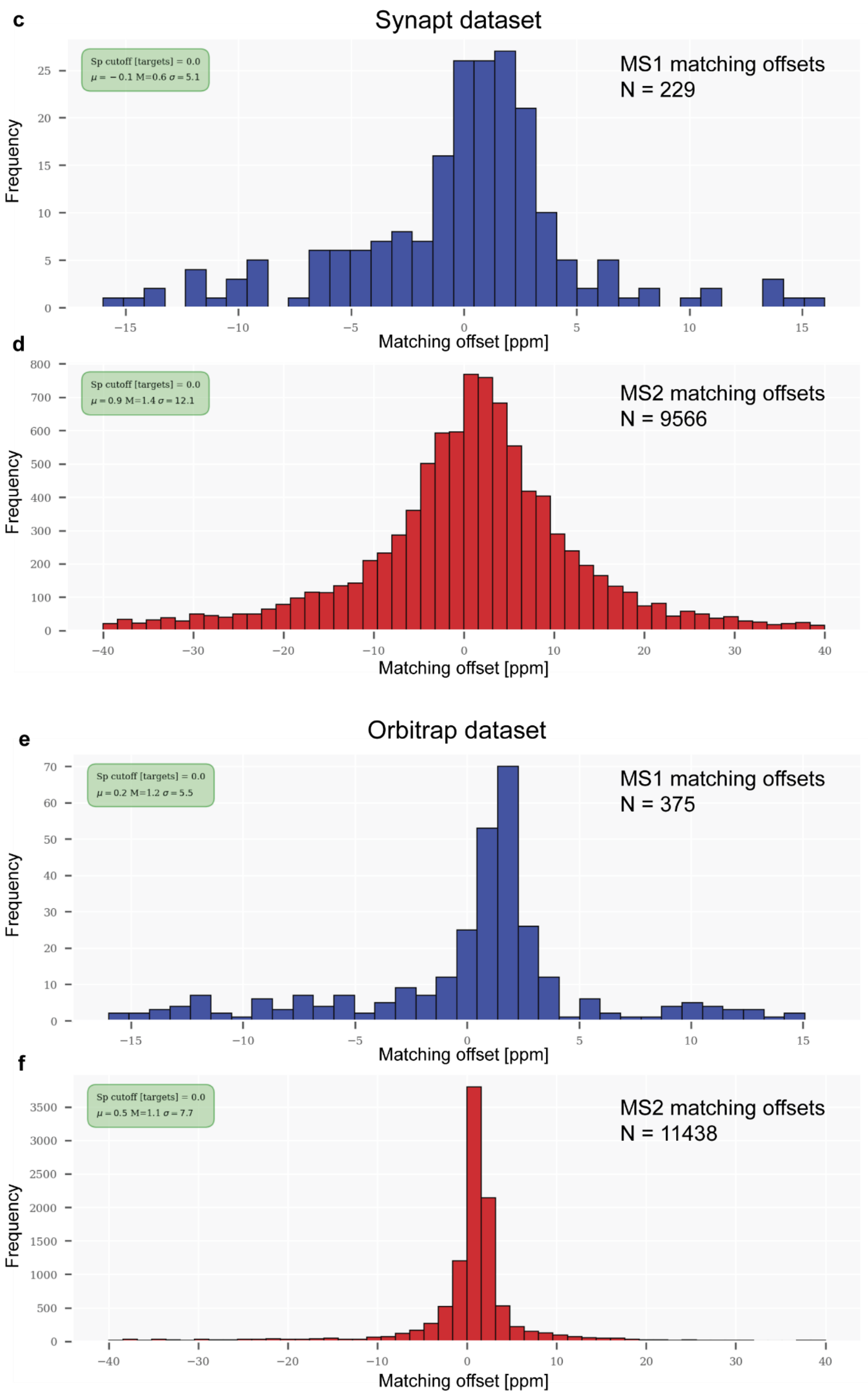
c
Synapt dataset
Sp cutoff [targets] = 0.0
μ = −0.1 M=0.6 σ = 5.1
MS1 matching offsets
N = 229
Frequency
Matching offset [ppm]
d
Sp cutoff [targets] = 0.0
μ = 0.9 M=1.4 σ = 12.1
MS2 matching offsets
N = 9566
Frequency
Matching offset [ppm]
e
Orbitrap dataset
Sp cutoff [targets] = 0.0
μ = 0.2 M=1.2 σ = 5.5
MS1 matching offsets
N = 375
Frequency
Matching offset [ppm]
f
Sp cutoff [targets] = 0.0
μ = 0.5 M=1.1 σ = 7.7
MS2 matching offsets
N = 11438
Frequency
Matching offset [ppm]

Supplementary Figure 18

Example of a tandem spectrum for a 13-mer synthetic RNA oligo. F is a one letter Pytheas notation and [Am] is an extended notation for 2'-OMe adenosine. Fragment ion matches are highlighted, color-coded based on their ion series and reported in the table below the spectrum using predicted m/z values. Unlike short oligomers, sequences >10-12 nt in length typically observe decreased number of MS2 matches (*n*/*L* ~0.4-0.6), that results in lower Sp scores and reduced rate of identification. In the example provided, none of the 9 predicted ion series get consecutive matches across the entire sequence (no sequence read-through observed), and at least 3 out of 9 series (a, b/x) are poorly represented with fragment ions.

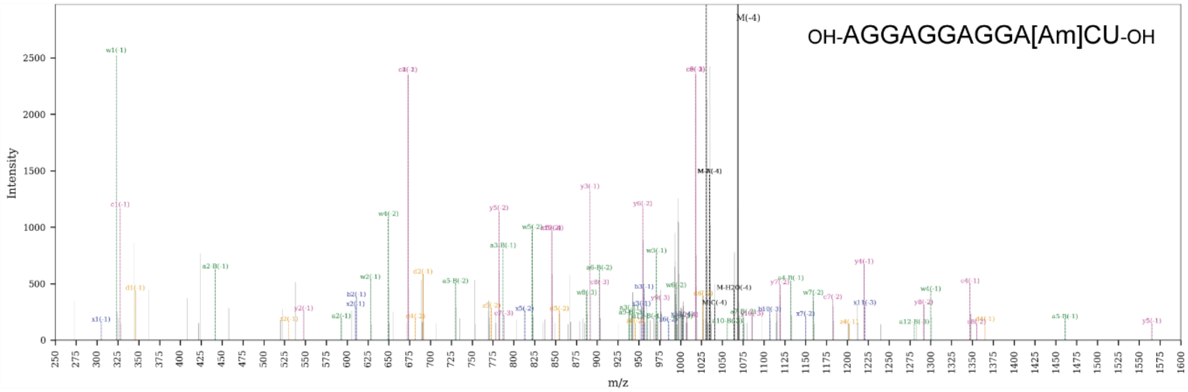

	a(-1)	a(-2)	a(-3)	a(-4)	a-B(-1)	a-B(-2)	a-B(-3)	a-B(-4)	b(-1)	b(-2)	b(-3)	b(-4)
A												
G	593.1258				442.0764				611.1364			
G	938.1732	468.5827			787.1238	393.058			956.1838	477.588		
A	1267.2258	633.109			1132.1713	565.5817			1285.2363	642.1142		
G	1612.2732	805.6327			1461.2238	730.108			1630.2838	814.638		
G		978.1564	651.7683			902.6317	601.4185			987.1617	657.7718	
A		1142.6827	761.4525			1075.1554	716.4343			1151.6879	767.456	
G		1315.2064	876.4683			1239.6817	826.1185			1324.2117	882.4718	
G		1487.7301	991.4841			1412.2054	941.1343			1496.7354	997.4876	
A		1652.2564	1101.1683	825.6243		1584.7291	1056.1501	791.8606		1661.2616	1107.1718	830.1269
F			1215.5244	911.3913			1170.5062	877.6277			1221.5279	915.8939
C			1317.2048	987.6516			1280.1904	959.8908			1323.2083	992.1543
U												

	c(-1)	c(-2)	c(-3)	c(-4)	d(-1)	d(-2)	d(-3)	d(-4)	w(-1)	w(-2)	w(-3)	w(-4)
A	328.0447				346.0553							
G	673.0921				691.1027						1342.188	1006.389
G	1018.1396	508.5659			1036.1501	517.5712					1227.1722	920.1272
A	1347.1921	673.0921			1365.2026	682.0974				1668.7385	1112.1564	833.8653
G	1692.2395	845.6158				854.6211				1504.2122	1002.4722	
G		1018.1396	678.4238			1027.1448	684.4273			1331.6885	887.4564	
A		1182.6658	788.1079			1191.6711	794.1115			1159.1648	772.4406	
G		1355.1895	903.1238			1364.1948	909.1273			994.6385	662.7564	
G		1527.7133	1018.1396			1536.7185	1024.1431		1645.2375	822.1148		
A		1692.2395	1127.8237	845.6158			1133.8273	850.1185	1300.19	649.5911		
F			1242.1798	931.3829			1248.1833	935.8855	971.1375	485.0648		
C			1343.8602	1007.6432			1349.8637	1012.1459	628.0693			
U									323.028			

	x(-1)	x(-2)	x(-3)	x(-4)	y(-1)	y(-2)	y(-3)	y(-4)	z(-1)	z(-2)	z(-3)	z(-4)
A												
G			1336.1845	1001.8864			1315.5326	986.3975			1309.529	981.8948
G			1221.1687	915.6245			1200.5168	900.1356			1194.5132	895.633
A		1659.7332	1106.1529	829.3627		1628.7553	1085.5009	813.8737		1619.75	1079.4974	809.3711
G		1495.2069	996.4687			1464.2291	975.8168			1455.2238	969.8132	
G		1322.6832	881.4529			1291.7053	860.801			1282.7001	854.7974	
A		1150.1595	766.4371			1119.1816	745.7851			1110.1763	739.7816	
G		985.6332	656.7529			954.6554	636.101			945.6501	630.0974	
G	1627.2269	813.1095			1565.2711	782.1316			1547.2606	773.1264		
A	1282.1795	640.5858			1220.2237	609.6079			1202.2131	600.6026		
F	953.1269	476.0596			891.1712	445.0817			873.1606	436.0764		
C	610.0588				548.103				530.0924			
U	305.0175											

C(-1)	A(-1)	U(-1)	G(-1)	F(-1)	M-C(-3)	M-C(-4)	M-A(-3)	M-A(-4)	M-U(-3)	M-U(-4)	M-G(-3)	M-G(-4)	M-F(-3)	M-F(-4)	M-H2O(-4)
110.0354	134.0467	111.0195	150.0416	134.0467	1388.2023	1040.8998	1380.1986	1034.897	1387.8743	1040.6538	1374.8669	1030.8982	1380.1986	1034.897	1064.158

Supplementary Table 1

Pseudouridine modifications identified in the *S. cerevisiae* 18S dataset. Only the highest scoring (light or heavy) target sequences are reported for each pseudouridine position. The complete list of known 18S modifications is obtained from Taoka *et al.*[1]

Position	Target sequence	S_p	ΔS_pD	Min FDR[a]
106	97-CUC[Am]UUAAA[Ψ]CAG-109	0.237	0.89	4%
120	116-UUUA[Ψ]UUG-123	1.757	0.82	<1%
211	205-UAUUUA[Ψ]UAG-214	0.885	0.82	<1%
302	[b]	–	–	
466	[c]	–	–	
632	[d]	–	–	
759	752-AAAAAAU[Ψ]AG-761	1.029	0.84	<1%
766	[b]	–	–	
999	998-A[Ψ]CAG-1002	0.74	0.27	<1%
1181	1180-C[Ψ]UAAUU[Ψ]G-1188[f]	0.514	0.06	2%
1187	1180-C[Ψ]UAAUU[Ψ]G-1188[f]	0.514	0.06	2%
1290	1289-U[Ψ]G-1291	0.578	N/A[e]	1%
1415	1413-UU[Ψ]G-1416	0.35	0.46	3%

[a] Lowest FDR threshold at which the modification is detected.

[b] Precursor ion was not picked for MS2 acquisition.

[c] [Ψ]G sequence is too short to be considered for MS2 identification.

[d] Precursor ion not detected.

[e] No competing decoy present.

[f] Identified based on a single observation.

Supplementary Table 2

Spectral identification coverage and FDR for the *S. cerevisiae* tRNA dataset.

mgf scans[a]	1874
Top targets[b]	813
Top decoys[b]	157
Unique target sequences[c]	344
Highest S_p score	1.977
Unique target sequences (10% FDR)[c]	280
Unique target sequences (5% FDR)[c]	182
Unique target sequences (1% FDR)[c]	76

[a] mgf scans refer to the amount of acquired MS2 spectra present in the input file.

[b] Top targets and decoys refer to rank 1 OSMs.

[c] Total number of targets with unique sequences. FDR threshold (if applied) is specified in the parenthesis.

Supplementary Table 3

Modification types identified using targeted MS/MS analysis performed on the mixture of *S. cerevisiae* tRNAs.

Modification	ID[a]	*m/z*	z	RT (min)	Assigned sequence[a]	S_p
5-carbamoylmethyluridine	[ncm5U]	839.11	2	32	A[mC]U[ncm5U]G	0.787
N4-acetylcytidine	[ac4C]	506.569	2	20.5	C[ac4C]G	0.705
Wybutosine	[yW]	1387.214	3	56.5	A[Cm]U[Gm]AA[yW]AU[mC]UG	0.743
5-methoxycarbonylmethyl-2-thiouridine	[mcm5s2U]	971.779	3	32.9	CU[mcm5s2U]UCACCG	0.942
5-methoxycarbonyluridine	[mcm5U] [t6A]	1344.177	3	34.9	ACU[mcm5U]CU[t6A]AUCAG	0.524
6-isopentenyladenosine	[i6A]	777.448	3	58.5	A[i6A]AUCUG	1.334
6-isopentenyladenosine	[i6A]	879.129	3	56.8	CA[i6A]AUCUG	1.512
6-isopentenyladenosine	[i6A]	981.465	3	48.7	UA[i6A]AUCUUG	1.534
6-isopentenyladenosine	[i6A]	1088.146	3	57.9	A[i6A]AUCUUU[Um]G	1.326
6-threonylcarbamoyladenosine	[t6A]	795.433	3	29.9	UU[t6A]AUCG	0.405
6-threonylcarbamoyladenosine	[t6A]	1006.796	3	27	CUUAU[t6A]ACG	0.325
6-threonylcarbamoyladenosine	[t6A]	1230.907	4	35.6	ACUCUU[t6A]AUCAUAAG	0.868
6-threonylcarbamoyladenosine	[t6A]	887.625	2	27.3	CU[t6A]AG	0.29
Dihydrouridine	[D]	945.115	2	19.2	[D]CUCCG	1.24

[a]Modification notations are the same as used in **Table 4** and **Supplementary Fig.6**.

Supplementary Table 4

Total sequence coverage and N1-methylpseudouridine ($m^1\Psi$) coverage for 3995-nt long RNA containing SARS-CoV-2 spike protein coding region. Data are shown for three independent samples containing ^{14}N-labeled (light), ^{15}N-labeled (heavy), or a nearly equimolar mixture of the two mRNA species (light + heavy).

	All sequence IDs		Unique sequence IDs only	
	All (%)[a]	$m^1\Psi$ (%)[b]	All (%)[a]	$m^1\Psi$ (%)[b]
RNase T1 dataset				
light	70.7	73.2	36.0	41.4
heavy	70.8	74.0	36.0	42.2
light + heavy	60.8	61.8	26.3	30.2
RNase A dataset				
light	48.0	22.6	5.2	1.8
heavy	48.7	22.9	5.6	2.1
light + heavy	47.6	22.4	5.0	1.8
T1 and A datasets combined				
light	86.9	79.3	39.9	43.0
heavy	87.4	80.4	41.6	44.3
light + heavy	80.1	69.4	30.4	31.8

[a] Percent of the total 3995 nt detected via Pytheas IDs

[b] Percent of the total 827 $m^1\Psi$ detected using Pytheas sequence IDs

Supplementary Table 5

Comparison of the publicly available software for analysis of RNA tandem MS data.

	Pytheas	NASE	Ariadne
Code availability	Freely available	Available as part of the OpenMS code	Not available
Code execution	Windows, Mac, Linux as GUI or command line	Windows, Mac, Linux as GUI or command line	Server only
Speed	Moderate	High	High
RNA modifications	Available	Available	Available
5’ and 3’ chemistry	User defined, flexible	Preset	User defined, flexible
Isotope Labeling	User specifies isotope composition for up to two RNA species.	None found	Some labeling schemes are available for a single RNA species only.
Scoring algorithm	Details provided. Can be adapted to accommodate changes in MS/MS fragmentation.	Poorly described	Contribution of factors is not well defined
Decoys and FDR	Available	Available	Not available
Sequence mapping for bottom-up applications	Available	Not available	Available
Other advantages	• Descriptive statistical plots and tools available to assist in the database matching process<br>• Support of custom cleavage specificities for input RNA	• Integrated into OpenMS, with complete automation of peak picking/filtering and peak integration for quantitative analysis<br>• Enables search for variable RNA modifications	• Easy to set-up<br>• Quick oligonucleotide calculator tool for precursor and fragment ion masses<br>• Enables search for variable RNA modifications

Supplementary Table 6

List of user-defined parameters applied to generate the *in silico* digest library for analysis of *E. coli* 16S datasets. In italic font are the names of the input files provided by the user.

Parameter	Value
Cleaving enzyme (RNAse)	T1
Missed cleavages	2
RNA molecule 5' chemistry	P, OH
RNA molecule 3' chemistry	P, OH
Nucleolytic fragments 5' chemistry	OH
Nucleolytic fragments 3' chemistry	P
Modification profile	*modfile_16S*
Unlabeled nucleotides elemental composition (light)	*nts_alphabet_light_std*
Labeled nucleotides elemental composition (heavy)	*nts_alphabet_heavy_std*
Ion mode	Negative
Minimum length fragment ions	3
CID series	a, a-B, b, c, d, w, x, y, z, y-P, z-P
MS1 level m/z window	400-2000
MS2 level m/z window	300-2000
Charge table precursor ions	Standard
Charge table fragment ions	Standard
Decoys	Yes
SeqX consolidation	Yes
MS1 SeqX threshold (ppm)	16
MS2 SeqX threshold (ppm)	40

Supplementary Table 7

Excerpt of the Pytheas elemental composition file with two (light and heavy) RNA species. Only composition of the nucleobase is shown here.

^{14}N-labeled (light)

One-letter ID	Extended ID	Parent nt	C	O	H	N	P	S	^{13}C	^{18}O	^{15}N	^{2}H
A	A		5	0	4	5	0	0	0	0	0	0
G	G		5	1	4	5	0	0	0	0	0	0
C	C		4	1	4	3	0	0	0	0	0	0
U	U		4	2	3	2	0	0	0	0	0	0
F	[Am]	A	5	0	4	5	0	0	0	0	0	0
g	[mmG]	G	7	1	8	5	0	0	0	0	0	0
h	[ho5C]	C	4	2	4	3	0	0	0	0	0	0
D	[D]	U	4	2	5	2	0	0	0	0	0	0
m	[mcm5s2U]	U	7	3	7	2	0	1	0	0	0	0

^{15}N-labeled (heavy)

One-letter ID	Extended ID	Parent nt	C	O	H	N	P	S	^{13}C	^{18}O	^{15}N	^{2}H
A	A		5	0	4	0	0	0	0	0	5	0
G	G		5	1	4	0	0	0	0	0	5	0
C	C		4	1	4	0	0	0	0	0	3	0
U	U		4	2	3	0	0	0	0	0	2	0
F	[Am]	A	5	0	4	0	0	0	0	0	5	0
g	[mmG]	G	7	1	8	0	0	0	0	0	5	0
h	[ho5C]	C	4	2	4	0	0	0	0	0	3	0
D	[D]	U	4	2	5	0	0	0	0	0	2	0
m	[mcm5s2U]	U	7	3	7	0	0	1	0	0	2	0

Supplementary Table 8

Basis for consolidation of sequences that cannot be uniquely identified via precursor or fragment ion masses. For example, if 5,6-D-uracil is used for labeling of the pyrimidines, and MS instrument accuracy is above 22.3 ppm (MS1) and 35 ppm (MS2), SeqX should be used at the step of *in silico* digestion to merge OH-ACG-P and OH-AΨG-P into OH-AXG-P by setting SeqX mass tolerance for precursor and fragment ions**.** SeqX is used to consolidate sequences of the same length, containing one or multiple residues that are similar by mass and are positional substitutes of one another.

	Unlabeled	5,6-D-uracil labeled
Precursor ions (MS1):		
m/z: OH-ACG-p (-1)	996.1440	998.1565
m/z: OH-AΨG-p (-1)	997.1280	998.1342
Δ*m/z*	0.9840	0.0223
Δppm	987.8	<u>22.3</u>
Fragment ions (MS2):		
m/z: OH-ACG-P : c2 (-1)	633.0860	635.0985
m/z: OH-AΨG-P : c2 (-1)	634.0699	635.0763
Δ*m/z*	0.9840	0.0223
Δppm	1551.7	<u>35.0</u>
m/z: OH-ACG-P : y2 (-1)	667.0915	669.1040
m/z: OH-AΨG-P : y2 (-1)	668.0755	669.0818
Δ*m/z*	0.9840	0.0223
Δppm	1472.9	<u>33.2</u>

Supplementary Table 9

95 RNA oligonucleotides used for training and validation of the Pytheas S_p score. Sequences are divided by oligonucleotide length, with modified nucleosides enclosed in square brackets. All oligonucleotides have 5'-OH and 3'-P chemistry. [Ψ] stands for pseudouridine and [Xm] for 2'-O-methylations.

3-mers	4-mers	5-mers	6-mers	7-mers	8-mers
ACG	GAAC	GGAAU	AAGAAC	GAGAAAC	AGAAAAAU
AGU	A[Cm]AG	CUUCG	AAUUUG	AUU[Ψ]CUG	AUUUUCAG
UCG	CUAG	AAUCG	UCCAAG	AAAAAUG	AACAAUUG
GAC	CCUG	AUCAG	CAUUUG	UCUCAAG	CACAAUCG
UAG	AAGC	CCACG	GAGGGC	AACUAUG	CCCCCUUG
	GAAU	CCUUG	UAAUCG	AAUCAUG	AACACCAG
	CAAG	AAAGU	GAGAGC	GGAAGAC	UUUCUCUG
	UCCG	GAAGU	CACAAG	CAACUCG	UCACCACG
	AUUG	AGAAU	ACCUCG	AGGAAAC	CUACAAUG
	UCUG	AAACG	AAUACG	AAUACCG	CACUCACG
	AAUG	UCUCG	UUCCCG	CCCCCUG	ACUCCAUG
	AGGC	CUCAG	GAAGGC		GGAGGAAU
	AGGU		GGAAAC		AAUUCCAG
	CUUG		GAAGAC		AUCUACUG
			AAACUG		

9-mers	10-mers	11-mers	12-mers	13-mers	14-mer
CCCUACUUG	UAUUUAUUAG	GAGAGGAGAGC	UUUAAUUCUUUG	UUUCCUUUUUCAG	UUAUCCACUACCAG
UUACCAUAG	UUCUAUUUUG	ACCUCAUAAAG	AGGAGGAGGA[Am]C	GAGAGGAGAAAGU	
CAUACAAAG	AGGAGAGGAC	AAAGG[Cm]AGAAU	ACUCACUUAAUG	U[Gm]CUACAUCUCAG	
AGGA[Um]GAGC	AAAGAGAAGC	AAUUAAACCUG	CAAAUACACUCG	CUUCUAUCCUC[Am]G	
CUCAUCAUG	CUCUUUCUUG				
	GG[Gm]AGAGGAU				

Supplementary References

1. Taoka, M. *et al.* The complete chemical structure of Saccharomyces cerevisiae rRNA: partial pseudouridylation of U2345 in 25S rRNA by snoRNA snR9. *Nucleic Acids Res* **44**, 8951-8961 (2016).
2. Addepalli, B., Venus, S., Thakur, P., Limbach, P.A. Novel ribonuclease activity of cusativin from Cucumis sativus for mapping nucleoside modifications in RNA. *Anal Bioanal Chem* **409**, 5645-5654 (2017).
3. Wein, S. *et al.* A computational platform for high-throughput analysis of RNA sequences and modifications by mass spectrometry. *Nat Commun* **11**, 926 (2020).
