# Supplementary material for: Pytheas: a software package for the automated analysis of RNA sequences and modifications via tandem mass spectrometry
Source: Nat Commun. 2022 May 3;13:2424. doi: 10.1038/s41467-022-30057-5 (PMC9065004; doi:10.1038/s41467-022-30057-5)
Supplement: Supplementary file 3 — Reporting Summary [file 41467_2022_30057_MOESM3_ESM.pdf]

nature portfolio

Corresponding author(s): James R. Williamson

Last updated by author(s): Mar 6, 2022

Reporting Summary

Nature Portfolio wishes to improve the reproducibility of the work that we publish. This form provides structure for consistency and transparency in reporting. For further information on Nature Portfolio policies, see our Editorial Policies and the Editorial Policy Checklist.

Statistics

For all statistical analyses, confirm that the following items are present in the figure legend, table legend, main text, or Methods section.

n/a	Confirmed	
☐	☒	The exact sample size (*n*) for each experimental group/condition, given as a discrete number and unit of measurement
☐	☒	A statement on whether measurements were taken from distinct samples or whether the same sample was measured repeatedly
☒	☐	The statistical test(s) used AND whether they are one- or two-sided<br>*Only common tests should be described solely by name; describe more complex techniques in the Methods section.*
☒	☐	A description of all covariates tested
☐	☒	A description of any assumptions or corrections, such as tests of normality and adjustment for multiple comparisons
☐	☒	A full description of the statistical parameters including central tendency (e.g. means) or other basic estimates (e.g. regression coefficient) AND variation (e.g. standard deviation) or associated estimates of uncertainty (e.g. confidence intervals)
☒	☐	For null hypothesis testing, the test statistic (e.g. *F*, *t*, *r*) with confidence intervals, effect sizes, degrees of freedom and *P* value noted<br>*Give P values as exact values whenever suitable.*
☒	☐	For Bayesian analysis, information on the choice of priors and Markov chain Monte Carlo settings
☒	☐	For hierarchical and complex designs, identification of the appropriate level for tests and full reporting of outcomes
☒	☐	Estimates of effect sizes (e.g. Cohen's *d*, Pearson's *r*), indicating how they were calculated

Our web collection on statistics for biologists contains articles on many of the points above.

Software and code

Policy information about availability of computer code

Data collection	MassHunter LC/MS Acquisition B.06.01; MassHunter Qualitative Analysis B.07.00; MassLynx V4.1, ProteinLynx Global Server V2.5.2; Xcalibur V4.3.73.11, MSConvert from ProteoWizard 3.0.11537.
Data analysis	Pytheas (https://github.com/ldascenzo/pytheas), Isodist (https://doi.org/10.1021/ac800080v)

For manuscripts utilizing custom algorithms or software that are central to the research but not yet described in published literature, software must be made available to editors and reviewers. We strongly encourage code deposition in a community repository (e.g. GitHub). See the Nature Portfolio guidelines for submitting code & software for further information.

Data

Policy information about availability of data

All manuscripts must include a data availability statement. This statement should provide the following information, where applicable:

- Accession codes, unique identifiers, or web links for publicly available datasets
- A description of any restrictions on data availability
- For clinical datasets or third party data, please ensure that the statement adheres to our policy

The mass spectrometry data and Pytheas output files generated in this study have been deposited in the ProteomeXchange Consortium database via the PRIDE repository:
The RNA training set is available under accession code PXD030435 [http://proteomecentral.proteomexchange.org/cgi/GetDataset?ID=PXD030435]
The E. coli 16S RNA dataset is available under accession code PXD030538 [http://proteomecentral.proteomexchange.org/cgi/GetDataset?ID=PXD030538]
The S. cerevisiae 18S RNA dataset is available under accession code PXD030563 [http://proteomecentral.proteomexchange.org/cgi/GetDataset?ID=PXD030563]

The S. cerevisiae tRNA dataset is available under accession code PXD030844
[http://proteomecentral.proteomexchange.org/cgi/GetDataset?ID=PXD030844]
The SARS-COV2 mRNA dataset is available under accession code PXD030845
[http://proteomecentral.proteomexchange.org/cgi/GetDataset?ID=PXD030845]

Additional databases used: MODOMICS [http://genesilico.pl/modomics], SGD [https://www.yeastgenome.org], GtRNAdb [http://gtrnadb.ucsc.edu].
Additional datasets used: LC-MS/MS analysis of human long ribosomal RNA [https://www.ebi.ac.uk/pride/archive/projects/PXD016323]

Source data are provided with this paper.

Field-specific reporting

Please select the one below that is the best fit for your research. If you are not sure, read the appropriate sections before making your selection.

☒ Life sciences ☐ Behavioural & social sciences ☐ Ecological, evolutionary & environmental sciences

For a reference copy of the document with all sections, see nature.com/documents/nr-reporting-summary-flat.pdf

Life sciences study design

All studies must disclose on these points even when the disclosure is negative.

Sample size	Except for the reference RNA(SupplFig 1,2), all other RNA LC-MS/MS datasets contain several hundreds of tandem spectra that were matched, scored, and statistically assessed using Pytheas tools. Here, sample size may vary and is determined by many factors including RNA sample, digestion, chromatography, MS instrument and acquisition parameters. Reference RNA dataset, used for training and validation of the search algorithm, was composed of 95 individual high-quality MS/MS spectra, that were manually curated and that could be ambiguously assigned to respective sequences. Oligonucleotides in size range of 3-13 nt are sufficiently presented with at least 4 individual sequences (SupplTable 9). Sequences over 14 nt were not considered, as they are very rare in most bottom-up RNA MS applications.
Data exclusions	For FDR calculations and statistical plots (Fig 3, SupplFig 1,2, 4) targets with competing decoys were used and targtes w/o decoys excluded.
Replication	All the data are reported as a single replicate. Since each LC-MS/MS dataset is represented by hundreds of tandem spectra and Pytheas matched sequence IDs, the major conclusions were derived from global distributions of data within any given dataset. Presence of individual modifications is based on at least two observations (e.g., light and heavy IDs, or multiple IDs across same chromatographic peak) unless otherwise stated (SupplTabl 1). Sequence coverage in SARS-CoV-2 mRNA has been reported for three mRNAs samples with different isotope composition (SupplTabl 4).
Randomization	N/A, as no separate groups of samples or datasets are reported
Blinding	N/A, as no separate groups of samples or datasets are reported

Reporting for specific materials, systems and methods

We require information from authors about some types of materials, experimental systems and methods used in many studies. Here, indicate whether each material, system or method listed is relevant to your study. If you are not sure if a list item applies to your research, read the appropriate section before selecting a response.

Materials & experimental systems

n/a	Involved in the study
☒	☐ Antibodies
☒	☐ Eukaryotic cell lines
☒	☐ Palaeontology and archaeology
☒	☐ Animals and other organisms
☒	☐ Human research participants
☒	☐ Clinical data
☒	☐ Dual use research of concern

Methods

n/a	Involved in the study
☒	☐ ChIP-seq
☒	☐ Flow cytometry
☒	☐ MRI-based neuroimaging
